# MONOPTEROS isoform MP11ir plays a role during somatic embryogenesis in *Arabidopsis thaliana*

**DOI:** 10.1093/plphys/kiaf602

**Published:** 2025-11-20

**Authors:** Barbara Wójcikowska, Samia Belaidi, Victoria Mironova, Sylvie Citerne, Hélène S Robert

**Affiliations:** Hormonal Crosstalk in Plant Development, Mendel Centre for Plant Genomics and Proteomics, CEITEC MU - Central European Institute of Technology, Masaryk University, Brno 625 00, Czech Republic; Institute of Biology, Biotechnology and Environmental Protection, Faculty of Natural Sciences, University of Silesia in Katowice, Katowice 40-032, Poland; Hormonal Crosstalk in Plant Development, Mendel Centre for Plant Genomics and Proteomics, CEITEC MU - Central European Institute of Technology, Masaryk University, Brno 625 00, Czech Republic; Laboratory of Functional Genomics and Proteomics, National Centre for Biomolecular Research, Faculty of Science, Masaryk University, Brno 625 00, Czechia; Department of Plant and Animal Biology, Radboud Institute for Biological and Environmental Sciences (RIBES), Radboud University, Nijmegen 6525 XZ, The Netherlands; INRAE, AgroParisTech, Institute Jean-Pierre Bourgin for Plant Sciences (IJPB), Université Paris-Saclay, Versailles 78000, France; Hormonal Crosstalk in Plant Development, Mendel Centre for Plant Genomics and Proteomics, CEITEC MU - Central European Institute of Technology, Masaryk University, Brno 625 00, Czech Republic

## Abstract

Auxin is crucial for plant morphogenesis, including embryo development. Exogenous auxin application is necessary for inducing embryogenic responses in *in vitro* cultured explants of Arabidopsis (*Arabidopsis thaliana*) and other plants. Thus, components of auxin transport, signaling, and metabolism are key to somatic embryogenesis. AUXIN RESPONSE FACTOR (ARF) transcription factors bind to auxin response elements to control auxin-responsive gene expression and are often repressed by AUXIN/INDOLE-3-ACETIC ACIDs (Aux/IAAs). MONOPTEROS (MP)/ARF5 is especially important in the embryogenic transition, being highly expressed during somatic embryogenesis; its mutant cannot develop somatic embryos. The *MP11ir* transcript, an alternatively spliced variant of *MP*, produces a truncated protein missing the Phox and Bem1p (PB1) domain, crucial for ARF–Aux/IAA dimerization. This renders MP11ir insensitive to Aux/IAA repression, suggesting auxin-independent regulation. High levels of *MP11ir* transcript are observed during auxin- and trichostatin-A-dependent induction of somatic embryogenesis. Both MP and MP11ir are essential for embryo regeneration in the *mpS319* mutant. However, overexpression of a truncated MP protein (ΔARF5) lacking the PB1 domain inhibits somatic embryogenesis, resulting in callus instead of somatic embryos. Overexpression of *ΔARF5*, lack of MP protein (*mp* mutant), or interference with MP action by the auxin-resistant BODENLOS (BDL) protein affects the expression of auxin biosynthesis genes. Our results suggest that these auxin-related genes might be targets of MP11ir and/or MP. Consequently, any adjustment to MP activity alters auxin homeostasis and endogenous auxin levels, hindering embryogenic transition.

## Teaser

In Arabidopsis, canonical and short AUXIN RESPONSE FACTOR 5/MONOPTEROS isoforms are required for somatic embryogenesis induction by regulating auxin biosynthetic *YUCCA* gene transcription.

## Introduction

Auxin plays a vital role in various aspects of plant development, including plant growth and morphogenesis in response to environmental signals through its transport, metabolism, and signaling (Caumon and Vernoux 2023; Cohen and Strader 2024; Liu et al. 2024). Recognized as a key trigger of developmental changes, auxin is a critical inducer of somatic embryogenesis (SE) in many plant species (Elhiti and Stasolla 2022). SE is a unique plant-specific developmental process that demonstrates the pluripotency of somatic cells and is widely exploited for *in vitro* plant regeneration. This technique is used in biotechnology for mass micropropagation of plants (Mehbub et al. 2022), plant biodiversity conservation (Ballesteros et al. 2024), and the production of transgenic plants (Lee and Wang 2023; Yang et al. 2024a; McFarland and Kaeppler 2025). With the rising global population, decreased crop yields due to climate change, and increasing environmental pollution, the importance of *in vitro* plant propagation techniques is growing (Krasteva et al. 2021; Hasnain et al. 2022). Understanding the molecular mechanisms of SE can enhance its efficiency and its optimization for recalcitrant species (Chen et al. 2022). Exogenous auxin treatment of explants modifies chromatin accessibility and the transcriptome of somatic cells (Wang et al. 2020; Wu et al. 2022). This modulation affects many genes, including those encoding transcription factors (TFs), proteins associated with hormone transport, metabolism, signaling, and stress response. The induction of SE is also associated with the expression of the genes encoding the core components of the nuclear auxin signaling pathway, such as AUXIN RESPONSE FACTORs (ARFs) and AUXIN/INDOLE-3-ACETIC ACIDs (Aux/IAAs) (Quintana-Escobar et al. 2019). Thus, the crosstalk between auxin and these transcriptional regulators is fundamental to the SE regulatory network.

MONOPTEROS (MP), known as AUXIN RESPONSE FACTOR 5 (ARF5), is pivotal in nuclear auxin signaling and has been identified in many plant species (Chandler 2016). The function of MP is well characterized, thanks to a wide range of tools like allelic mutants, overexpressing, and reporter lines in Arabidopsis (*Arabidopsis thaliana*), allowing for comprehensive functional, genetic, and microscopic analyses (Hardtke et al. 2004; Schlereth et al. 2010; Rademacher et al. 2011; Odat et al. 2014; Wu et al. 2015; Wójcikowska et al. 2023). Knowledge of the MP action is being transferred to commercially important species (Liu et al. 2018a; Xu et al. 2019; Yue et al. 2020). MP is crucial for many aspects of *in vivo* plant development, including flower, ovule, and pollen development (Wu et al. 2015; Liu et al. 2018b; Cucinotta et al. 2021), zygotic embryogenesis (Möller et al. 2017), root and shoot meristem establishment (Cole et al. 2009; Schlereth et al. 2010; Krogan et al. 2016), organ polarity maintenance (Bhatia et al. 2016), and vascularization patterns (Przemeck et al. 1996; Mattsson et al. 2003). MP is also critical for *in vitro* plant morphogenesis. *MP* is essential for an effective SE process because its expression is stimulated during SE; it is active in regions (cotyledons and shoot apical meristem [SAM]) where somatic embryos emerge; and, more importantly, explants of *mp* mutant cannot produce somatic embryos (Wójcikowska and Gaj 2017).

The MP protein has four distinct domains: a B3-type DNA-binding domain (DBD), dimerization domains (DD1 and DD2), a middle region (MR) region, and a Phox and Bem1 (PB1) domain (Guilfoyle 2015). MP binds to the AuxRE cis-elements in the promoter of target genes. As a TF, MP primarily acts as a positive regulator of gene expression (Boer et al. 2014; Freire-Rios et al. 2020). In Arabidopsis, MP binds as a dimer to AuxRE motifs organized as inverted repeats (IR), direct repeats (DR), and everted repeats (ER). IR consists of two AuxREs on opposite DNA strands facing each other, DR has two consecutive AuxREs on the same DNA strand, and ER features two AuxREs on opposite directions and DNA strands. The DD1 and DD2 domains flank the DBD, acting as a caliper for binding to IR motifs. For docking on ER and DR motifs, the MP DBD dimer relies on the MR or PB1 domains (Cancé et al. 2022). Additionally, AuxRE elements in the *MP* promoter indicate that its expression is regulated by cellular auxin level (Lau et al. 2011).

Three scenarios outline MP action. First, at low auxin levels, MP interacts with BODENLOS/INDOLE-3-ACETIC ACID 12 (BDL/IAA12) via PB1 domains, recruiting TOPLESS/TOPLESS RELATED (TPL/TPR), transcriptional co-repressor proteins, and HDA19 histone deacetylase enzyme. HDA19 removes the acetyl group from the histone H3 and H4 tails, condensing the chromatin and blocking the expression of auxin-responsive genes (Chandler 2016). In the presence of auxin, BDL is recruited by the SCF^TIR/AFBs^ complex, ubiquitinated, and degraded by the proteasome, releasing MP to form a complex with SWI/SNF chromatin remodeling ATPases BRAHMA (BRM) and SPLAYED (SYD). It unlocks the chromatin to provide histone acetyltransferases (HATs) and TFs access to the cis*-*elements of MP-regulated promoters, thus regulating gene transcription (Wu et al. 2015). Second, above-threshold auxin levels may potentially cause MP protein oligomerization with itself or other ARFs, inhibiting the expression of auxin-responsive genes, as observed *in vitro* (Korasick et al. 2014; Nanao et al. 2014) and *in vivo* for ARF19 (Powers et al. 2019). This scenario is plausible as plants with *MP* constitutive overexpression (Hardtke et al. 2004) phenocopy *mp* mutants (Przemeck et al. 1996). Third, the MP11ir isoform, produced by alternative splicing (AS) of the *MP* transcript during ovule and root development, lacks the PB1 domain due to the retention of intron 11, resulting in the translation of a truncated protein. This isoform is insensitive to Aux/IAA repression and could function independently of auxin. Ectopic expression of *MP11ir* partially complements *mp* mutant phenotypes during reproductive development, indicating that some MP functions do not require interaction with Aux/IAA repressors. MP11ir activates target genes in the ovule and root regions with low and high auxin concentrations (Cucinotta et al. 2021; Cavalleri et al. 2024).

To understand the role of MP11ir in the embryogenic transition, we analyzed its transcript level in Arabidopsis explants cultured *in vitro* and treated with SE inducers: auxins 2,4-dichlorophenoxyacetic acid (2,4-d), 1-naphthaleneacetic acid (NAA), and indole-3-acetic acid (IAA), and trichostatin A (TSA). High *MP11ir* transcript levels were associated with auxin- and TSA-dependent SE inductions. MP11ir can partially rescue the *mp* phenotype, improving its capacity for embryogenic transition. However, both MP and MP11ir are required to fully complement the *mp* phenotype. Overexpression of the truncated MP protein (ΔARF5) inhibits SE and alters the expression of auxin biosynthetic genes of the indole-3-pyruvic acid (IPyA) pathway: *TRYPTOPHAN AMINOTRANSFERASE OF ARABIDOPSIS 1* (*TAA1*), *TAA1-RELATED 1* (*TAR1*), *YUCCA 3* (*YUC3*), *YUC5*, and *YUC8*. This modulation changes endogenous auxin levels, negatively impacting embryogenic transition. We hypothesized that MP11ir may directly control *TAA1*, *YUC3*, *YUC5*, and *YUC8*. These findings suggest that MP11ir is involved in SE induction alongside the canonical MP, contributing to the regulation of auxin biosynthesis and homeostasis during this process.

## Results

### The presence of *MP11ir* transcript is specific for auxin-dependent embryogenic transition

**Figure 1.**
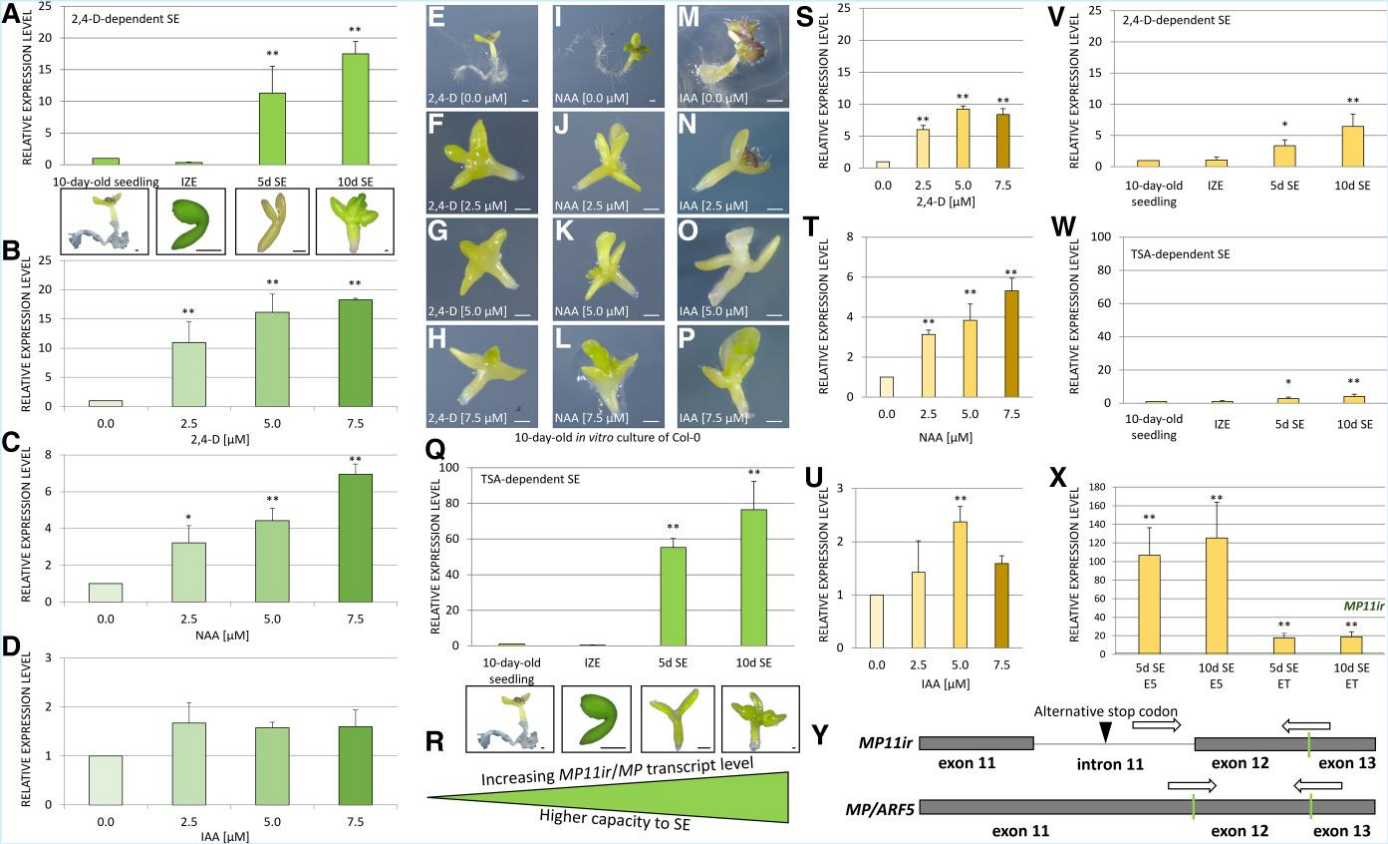
*MP11ir* and *MP* are expressed during somatic embryogenesis induction. **A)** The level of *MP11ir* transcript in IZE explants and during auxin-dependent (5.0 *µ*M 2,4-d) SE induction (5th and 10th day of *in vitro* culture). Values significantly different from the control culture (10-day-old seedlings) are marked with asterisks. The relative expression level was normalized to the internal control (*AT4G27090*) and calibrated to the expression in 10-day-old seedlings. A Student's *t*-test analysis was used to determine any values that were significantly different from 10-day-old seedlings (*P < 0.05; **P < 0.01) (*n* = 3; means ± SD are presented). The images were digitally extracted to illustrate the type of samples. **B-P)** The level of *MP11ir* transcript in the IZE explants cultured for ten days on medium without exogenous auxin, control condition (**E, I, M**), and treated with 2,4-d (**B**), NAA (**C**) and IAA (**D**) concentrations of 2.5 (**F, J, N**), 5.0 (**G, K, O**), and 7.5 *µ*M (**H, L, P**). Values significantly different from the control culture (explants untreated with 2,4-d, NAA, or IAA) are marked with asterisks. The relative expression level was calibrated to the 10-day-old IZE control culture, untreated with exogenous auxin. A Student's *t*-test analysis was used to determine any values that were significantly different from untreated IZE explants (*P < 0.05; **P < 0.01) (*n* = 3; means ± SD are presented). Scale bars indicate 1 mm (**E-P**). **Q)** The *MP11ir* transcript level in IZE explants and during TSA-dependent (1.0 *µ*M TSA) SE induction (5th and 10th day of *in vitro* culture). Values significantly different from the control culture (10-day-old seedlings) are marked with asterisks. A Student *t*-test analysis was used to determine any values that were significantly different from 10-day-old seedlings (*P < 0.05; **P < 0.01) (*n* = 3; means ± SD are presented). The images are identical to panel A for illustrating the type of samples. **R)** Together with data presented in Supplementary Fig. S2, we hypothesize a positive correlation between *MP11ir*, *MP* levels, and SE capacity. **S-U)** The level of *MP* transcript in the IZE explants cultured on medium without exogenous auxin and treated with 2,4-d (**S**), NAA (**T**), and IAA (**U**) concentrations of 2.5, 5.0, and 7.5 *µ*M. Values significantly different from the control culture (explants untreated with 2,4-d, NAA, or IAA) are marked with asterisks. The relative expression level was calibrated to the 10-day-old IZE control culture untreated with exogenous auxin. A Student's *t*-test analysis was used to determine any values that were significantly different from untreated IZE explants (*P < 0.05; **P < 0.01) (*n* ≥ 3; means ± SD are presented). **V, W)** The expression profile of *MP* in IZE explants and during auxin-dependent (5.0 *µ*M 2,4-d) (**V**) and TSA-dependent (1.0 *µ*M TSA) (**W**) SE induction (5th and 10th days of *in vitro* culture). Values significantly different from the expression of 10-day-old seedlings are marked with asterisks. A Student's *t*-test analysis was used to determine any values that were significantly different from 10-day-old seedlings (*P < 0.05; **P < 0.01) (*n* ≥ 3; means ± SD are presented). **X)** The expression profile of *MP* during auxin-dependent (E5) and TSA-dependent (ET) SE induction (5th and 10th days of *in vitro* culture). The relative expression level was calibrated to the *MP11ir* expression on the same day of *in vitro* culture. Values significantly different from the *MP11ir* expression are marked with asterisks. A Student's *t*-test analysis was used to determine any values that were significantly different from 10-day-old seedlings (*P < 0.05; **P < 0.01) (*n* ≥ 3; means ± SD are presented). **Y)** The location of primers used to detect *MP11ir* and *MP* transcripts is indicated. 2,4-d—2,4-dichlorophenoxyacetic acid; IAA—indole-3-acetic acid; IZE—immature zygotic embryo; NAA—1-naphthaleneacetic acid; SE—somatic embryogenesis; TSA—trichostatin A.

*MP11ir* was first detected in ovules, where it likely regulates target genes in cells with low auxin concentrations (Cucinotta et al. 2021). Given the critical role of auxin signaling proteins in the embryogenic transitions, we investigated whether *MP11ir* would be active during SE induced by exogenously applied synthetic auxin 2,4-d. We hypothesized that if *MP11ir* regulates its target genes in cells with low auxin concentrations, its presence would be minimal in cells undergoing embryogenic reprogramming, as those cells accumulate exogenous 2,4-d and endogenous IAA (Wójcikowska and Gaj 2015; Schulz and Segobye 2016). To test this, we analyzed *MP11ir* transcript levels in various samples: 10-day-old seedlings, flowers, leaves, immature zygotic embryos (IZEs), and IZE explants during auxin-dependent SE induction. *MP11ir* was not detected in leaves but was present in other tissues, notably flowers, as previously observed (Supplementary Fig. S1) (Cucinotta et al. 2021). Quantitative analysis revealed that *MP11ir* was most abundant during the auxin-dependent embryogenic transition, with 11 to 18 times higher expression than in seedlings (Fig. 1A). It suggests that *MP11ir* may regulate target gene expression in both low (Cucinotta et al. 2021) and high auxin concentrations (our results; Cavalleri et al. 2024), indicating a role in auxin-independent transcription control during SE.

### The presence of the *MP11ir* transcript depends on the type and concentration of exogenously applied auxin

To investigate the correlation between *MP11ir* transcript levels and auxin presence, we treated IZEs with various auxins, i.e. 2,4-d, NAA, and IAA, at increasing concentrations (0, 2.5, 5.0, and 7.5 *µ*M) for ten days. Different auxins at the same concentrations had varied effects on *MP11ir* levels. We observed a positive correlation between *MP11ir* levels and increasing concentrations of 2,4-d (Spearman correlation coefficient 0.87; *P* < 0.01), NAA (Spearman correlation coefficient 0.92; *P* < 0.01), but not IAA (Fig. 1B to D). The application of 2,4-d had the most substantial promoting effect on *MP11ir* levels. A 2.5 *µ*M concentration of 2,4-d increased *MP11ir* transcript levels 10-fold compared to untreated explants, while a 7.5 *µ*M concentration of 2,4-d (three times higher) resulted in an 18-fold increase (Fig. 1B, E to H). The highest concentration of NAA (7.5 *µ*M) only increased *MP11ir* levels sevenfold (Fig. 1C, I to L). IAA did not significantly affect *MP11ir* transcript levels (Fig. 1D, M to P). The strength of SE induction also varied with the type of applied auxins (Supplementary Fig. S2). In Arabidopsis IZE culture, 2,4-d is the most effective for SE induction (Supplementary Fig. S2A, D to G), followed by NAA (Supplementary Fig. S2B, H to K), with IAA being the least effective (Supplementary Fig. S2C, L to O). We found a positive correlation between the increase of *MP11ir* transcript levels and the strength of SE induction for 2,4-d (Spearman correlation coefficient 0.80; *P* < 0.01) and NAA (Spearman correlation coefficient 0.75; *P* < 0.01), but not for IAA. This data suggests that the type and concentration of auxin influence *MP11ir* levels and subsequently affect the efficiency of the SE process.

### The presence of *MP11ir* transcript is specific for the embryogenic transition independently of the presence of auxin

Synthetic auxin 2,4-d is the most common and effective inducer of SE in Arabidopsis and other plants (Wójcik et al. 2020). To determine whether *MP11ir* is specific for embryogenic transition rather than merely responding to the application of exogenous auxins, which may also act as stressors (Karami et al. 2023a), we assessed the impact of TSA, an inhibitor of histone deacetylases (HDAC) known to induce SE (Tanaka et al. 2008), on the levels of *MP11ir* transcript. IZE explants treated with 1 *µ*M TSA rapidly and massively regenerate somatic embryos (Wójcikowska et al. 2018). We measured *MP11ir* levels on the 5th and 10th days of TSA-dependent SE induction and found significantly elevated levels of *MP11ir*, 55 and 84 times higher than in seedlings, respectively (Fig. 1Q). These findings suggest that MP11ir as well as MP are specifically associated with the developmental switch from somatic to embryonic cell fate, highlighting their role in the embryonic transition (Fig. 1R).

### The *MP* transcript is detected during the SE process

The transcript encoding the canonical MP protein was also detected during the SE process, which is consistent with current knowledge (Wójcikowska and Gaj 2017). Its expression pattern was very similar to that of *MP11ir*. The *MP* transcript significantly accumulated in 2,4-d and NAA-treated IZE explants (all concentrations tested) and in explants treated with 5.0 *µ*M IAA (Fig. 1S to U). We observed a significant up-regulation of *MP* expression during auxin-dependent (Fig. 1V) and TSA-dependent (Fig. 1W) induction of the SE process. During auxin- and TSA-induced SE, *MP* is predominantly synthesized over *MP11ir* (Fig. 1X). During the auxin-induced embryogenic transition, the transcript level of *MP* was more than 100-fold higher than that of *MP11ir*, whereas it was nearly 18-fold higher during TSA-induced SE (Fig. 1X). These results indicate that both *MP* and *MP11ir* (Fig. 1Y) transcripts are synthesized during SE, denoting their involvement in the embryogenic transition.

### Both MP and MP11ir are necessary to fully rescue the SE-related phenotypes of the *mpS319* mutant

**Figure 2.**
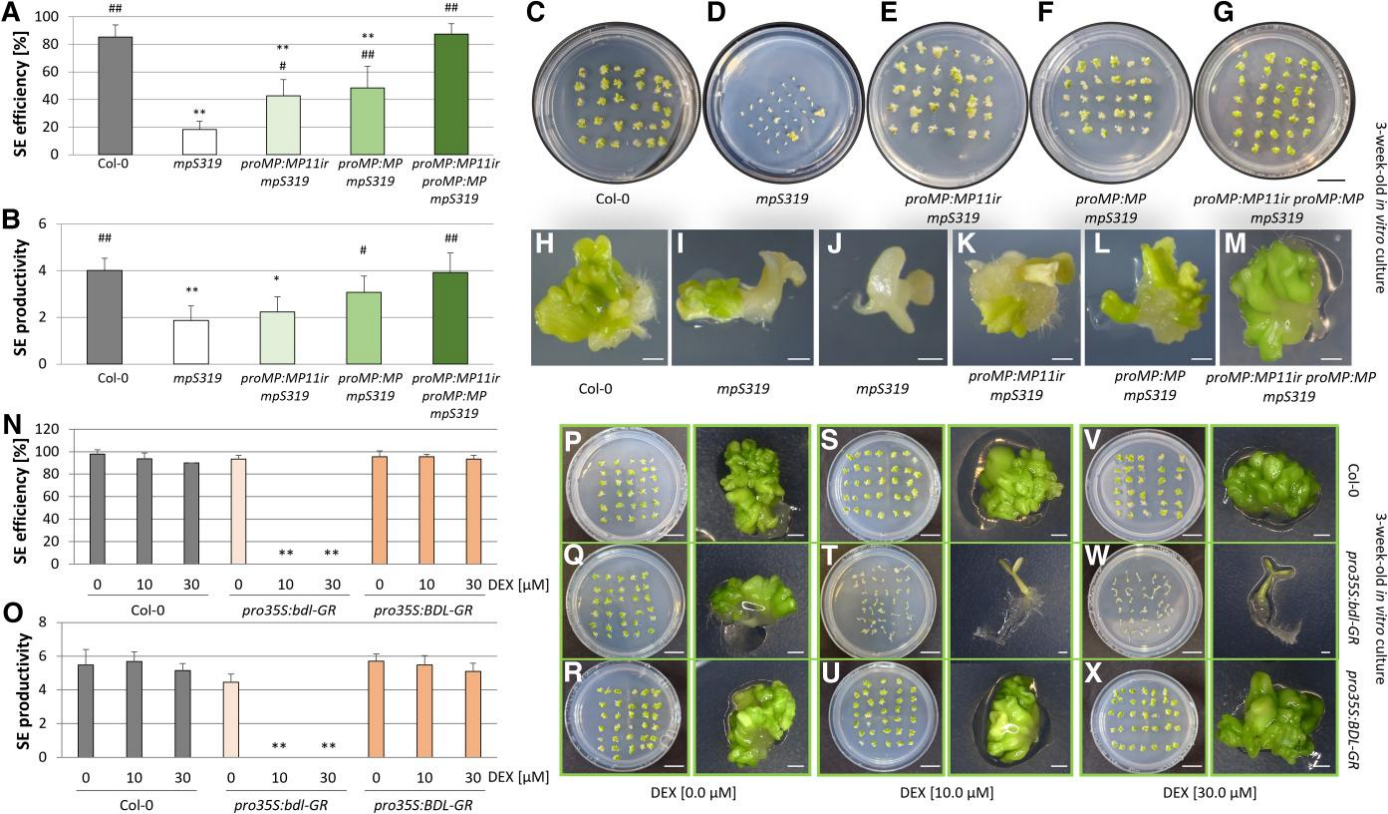
MP and MP11ir are both required to rescue the reduced SE capacity of *mpS319*. **A-M)** The embryogenic capacity (**A, B**) of the Col-0 (**C, H**), *mpS319* mutant (**D, I, J**), *proMP:MP11ir* (**E, K**), *proMP:MP* (**F, L**) and *proMP:MP11ir proMP:MP* (**G, M**) transgenes in *mpS319* background on the E5 medium evaluated in a 21-day-old culture. Values significantly different from the control culture (Col-0) are marked with asterisks (*), and from *mpS319* mutant are marked with hashtags (#). A Student's *t*-test analysis was used to determine any values that were significantly different (*^, #^*P* < 0.05; **^, # #^*P* < 0.01) (*n* ≥ 3; means ± SD are given). **N-X)** The embryogenic capacity (**N, O**) of the Col-0 (**P, S, V**), *pro35S:bdl-GR* (**Q, T, W**), *pro35S:BDL-GR* (**R, U, X**) transgenic lines on the E5 medium (**P, Q, R**) and E5 medium supplemented with 10 *µ*M (**S, T, U**) and 30 *µ*M (**V, W, X**) of DEX. SE capacity was evaluated in 21-day-old cultures. Values significantly different from the control culture (Col-0), untreated with DEX, are marked with asterisks (*). A Student *t*-test analysis was used to determine any values that were significantly different (*P < 0.05; **P < 0.01) (*n* = 3; means ± SD are presented). Scale bars indicate 1 mm for explant images and 1 cm for plate images. DEX—dexamethasone; SE—somatic embryogenesis.

Mutations in the *MP* gene dramatically reduce the capacity of IZE explants for SE. The *mpS319* mutant showed impaired SE efficiency (20%) and productivity (2 somatic embryos per explant) compared to Col-0 (85% efficiency and 4 embryos per explant) (Fig. 2A to D, H to J). Mutations in *MP* affect zygotic embryo development, potentially impacting the capacity of the IZE to undergo SE. To address this, we inhibited the MP-dependent signaling pathway by expressing an auxin-insensitive BDL/IAA12 (bdl) protein in the IZE. Overexpression of dexamethasone (DEX)-induced *bdl* in IZE completely abolished the embryogenic transition. Explants of the *pro35S:bdl-GR* line treated with DEX did not respond to the 2,4-d SE inducer and developed into seedlings. In contrast, Col-0 and the *pro35S:BDL-GR* transgenic line expressing a native BDL protein that is ubiquitinated and degraded by the proteasome in the presence of auxin, regenerated somatic embryos massively and independently of the presence of DEX (Fig. 2N-X). These results confirm the findings obtained with the *mpS319* line.

It was previously shown that MP11ir could partially rescue the *mpS319* mutant phenotypes during ovule development (Cucinotta et al. 2021). To investigate the function of MP11ir and its involvement in the SE process, we performed a complementation assay by expressing *MP11ir* or/and *MP* under the control of the *MP* promoter (*proMP:MP11ir-GFP*, hereafter *proMP:MP11ir,* and *proMP:MP* transgenes) in the *mpS319* mutant. The presence of MP11ir improved the SE capability of the *mpS319* mutant by 2.3-fold (Fig. 2A, B, E, and K). Interestingly, canonical MP did not fully rescue the *mpS319* SE defects, as only 48% of explants responded to SE induction in *proMP:MP mpS319* explants (Fig. 2A, B, F, and L). Both *MP* and *MP11ir* transcripts must be present to fully rescue the *mpS319* phenotype (Fig. 2A, B, G, and M). The complementation assay showed that MP11ir and MP are required together to fully rescue the *mpS319* SE defects and that both the canonical and truncated MP proteins are necessary for effective somatic embryo formation.

### MP and MP11ir localization during the SE process

**Figure 3.**
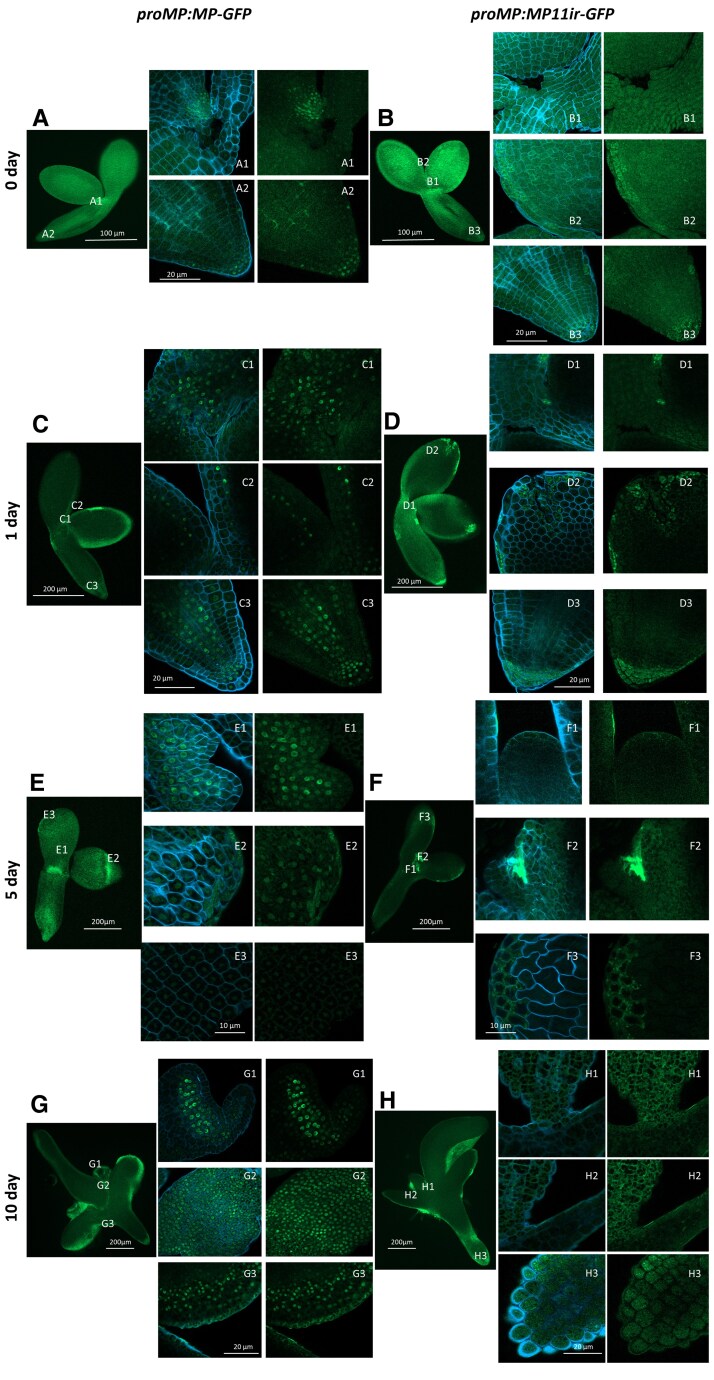
Expression pattern of MP and MP11ir during SE induction. Spatio-temporal localization of MP (**A**) and MP11ir (**B**) in IZE explants and explants cultured on the SE induction medium with 5 *μ*M 2,4-d by 1 (**C, D**), 5 (**E, F**) and 10 (**G, H**) days of SE culture. IZE explants were cultured from *proMP:MP-GFP* (**A, C, E, G)** and *proMP:MP11ir-GFP* (**B, D, F, H**) transgenic lines. The GFP fluorescence is shown in green. The blue fluorescence marks the cell (Renaissance staining). Magnification views (**A-H** 1 to 3) of the areas marked in **A-H**. Scale bars indicate 100 *μ*m (**A, B**), 200 *μ*m (**C-F**), 20 *μ*m (A1-D3, G1-H3), 10 *μ*m (E1-F3). 2,4-d—2,4-dichlorophenoxyacetic acid; IZE—immature zygotic embryo; SE—somatic embryogenesis.

To detect the MP and MP11ir protein during the auxin-dependent SE process, we used the transgenic lines *proMP:MP-GFP* and *proMP:MP11ir-GFP*. The MP signal was detected at different stages of SE induction (0, 1, 5, 10 days of culture) as a strong nuclear signal in the SAM, cotyledons, and root tip (Fig. 3A, C, E, and G). Notably, the MP nuclear signal progressively intensified and became widespread as the SE ages. In contrast, only faint and diffuse fluorescence signal was detected for MP11ir at all stages of culture (Fig. 3B, D, F, and H). The signal often lacks a clear nuclear localization pattern as observed for MP-GFP. This suggests that MP11ir protein is either (i) expressed below detection levels, (ii) is not expressed in these tissues under our experimental conditions, or (iii) subjected to post-transcriptional or -translational modifications resulting in the absence of a clear nuclear GFP signal. Using available lines, only MP protein was present during the SE process, suggesting that MP and MP11ir might have distinct roles during the SE process.

### Overexpression of a truncated MP inhibits the SE process

**Figure 4.**
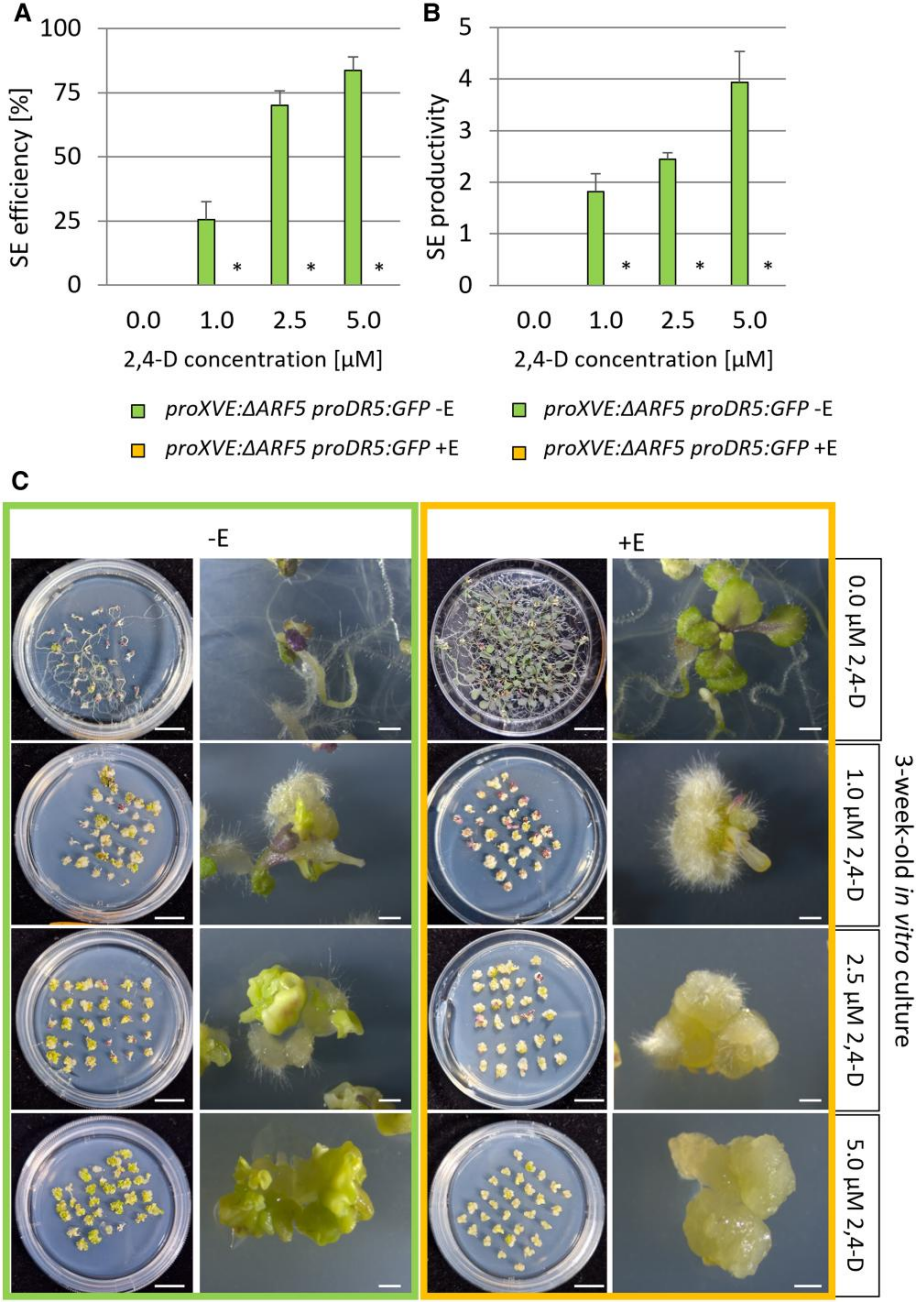
Overexpressing *ΔARF5* impairs SE induction. **A, B)** The embryogenic capacity of *proXVE:ΔARF5 proDR5:GFP* explants treated with different 2,4-d concentrations was evaluated in a 21-day-old culture. The significantly impaired SE efficiency (**A**) and productivity (**B**) of the *ΔARF5*-expressing explants are presented. **C)** Explants overexpressing *ΔARF5* and cultured on auxin-containing media could not regenerate somatic embryos and produced callus. *ΔARF5* overexpression was induced with 1 *µ*M estradiol (+E). Values significantly different from the control culture (Col-0) are marked with asterisks. A Student *t*-test was used to determine any values that were significantly different (*P < 0.05; *n* = 3; means ± SD are given). Scale bars indicate 1 mm for explant images and 1 cm for plate images. 2,4-d—2,4-dichlorophenoxyacetic acid; SE—somatic embryogenesis.

To better understand the complex roles of full-length and truncated MP proteins in SE, we examined the effects of ectopic expression of the truncated MP protein lacking the PB1 domain (ΔARF5), as MP11ir. We utilized a β-estradiol inducible *proXVE:ΔARF5 proDR5:GFP* transgenic line to overexpress *ΔARF5*. We observed that overexpression of *ΔARF5* inhibits the SE process when explants are cultured on a 2,4-D-supplemented medium (Fig. 4A and B). Specifically, explants overexpressing *ΔARF5* treated with 1 *µ*M β-estradiol and different concentrations of 2,4-d (1.0, 2.5, 5.0 *µ*M 2,4-d) developed callus tissue rather than somatic embryos (Fig. 4C). This finding underscores that constitutive overexpression of *ΔARF5* affects morphogenesis and disrupts the transition to embryonic development.

### Truncated and canonical MP proteins influence genes involved in auxin biosynthesis

**Figure 5.**
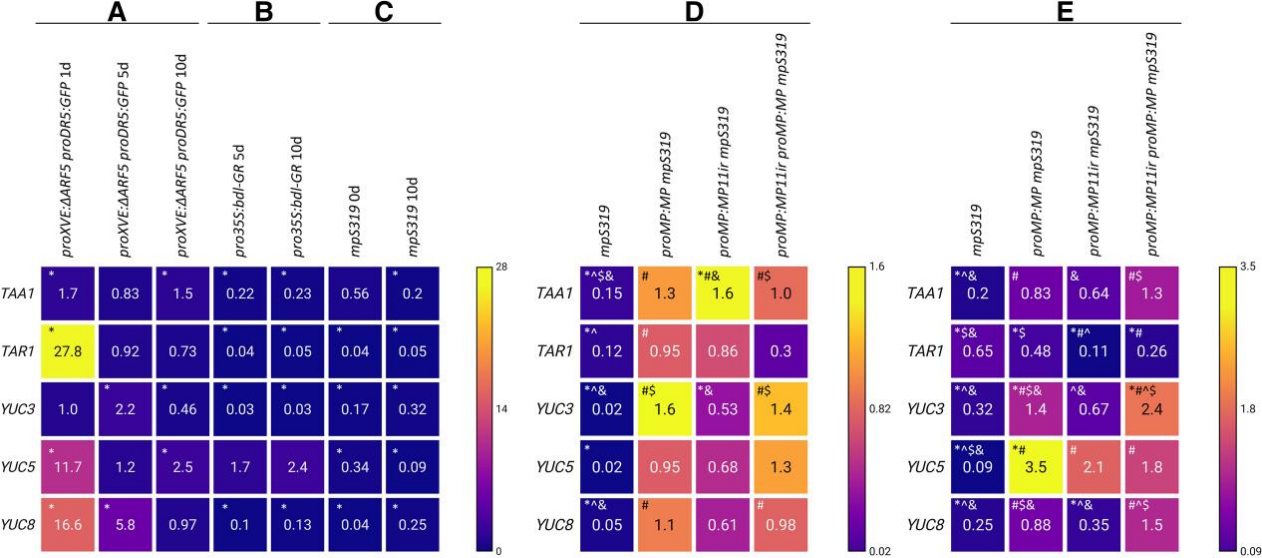
*TAA1*, *TAR1*, *YUC3*, *YUC5,* and *YUC8* expression levels are altered when MP-dependent transcriptional activities are impaired. **A-C)** Heatmap representing relative expression level of *TAA1*, *TAR1*, *YUC3*, *YUC5,* and *YUC8* auxin biosynthetic genes in the 1-, 5-, and 10-day-old embryogenic cultures of the *proXVE:ΔARF5 proDR5:GFP* transgenic line treated with 1 *µ*M β-estradiol (+E) (**A**), 5- and 10-day-old embryogenic cultures of the *pro35S:bdl-GR* transgenic line treated with 10 *µ*M dexamethasone (+DEX) (**B**), and 0- and 10-day-old (**C**) embryogenic cultures of *mpS319* mutant. The relative expression level was normalized to internal control (*AT4G27090*) and calibrated to the *proXVE:ΔARF5 proDR5:GFP* culture of the same age and untreated with 1 *µ*M β-estradiol (−E) (**A**) or *pro35S:bdl-GR* culture of the same age and untreated with 10 *µ*M dexamethasone (−DEX) (**B**), or Col-0 embryogenic culture of the same age (**C**). Values significantly different from the *proXVE:ΔARF5 proDR5:GFP* (−E) (**A**), *pro35S:bdl-GR* (−DEX) (**B**) or Col-0 (**C**) culture of the same age are marked with asterisks (Student's *t*-test; *P < 0.05; *n* = 3 ± SD). **D, E)** Heatmap showing relative expression level of *TAA1*, *TAR1*, *YUC3*, *YUC5,* and *YUC8* auxin biosynthetic genes in 5- (**D**) and 10-day-old (**E**) embryogenic cultures of *mpS319* mutant, *proMP:MP mpS319*, *proMP:MP11ir mpS319*, and *proMP:MP11ir proMP:MP mpS319* transgenic lines. Values significantly different (Student's *t*-test; *P* < 0.05; *n* = 3 ± SD) from the Col-0 culture of the same age are marked with asterisks (*), *mpS319* are marked with hashtags (#), *proMP:MP mpS319* are marked with carets (^), *proMP:MP11ir mpS319* are marked with dollar sign ($), *proMP:MP11ir proMP:MP mpS319* are marked with ampersands (&). Source data are presented in Supplementary Table S1.

The inhibitory effect of *ΔARF5* overexpression on the SE process prompted us to investigate its potential influence on endogenous auxin levels. SE inhibition often correlates with suboptimal auxin concentrations during SE induction with IZE. Indeed, overexpression of the *LEAFY COTELYDON 2* (*LEC2*) gene, a master regulator of the embryogenic process during auxin-dependent SE induction, resulted in SE inhibition due to increased endogenous auxin levels (Wójcikowska et al. 2013; Wójcikowska and Gaj 2015). Similarly, the combined treatment of exogenous auxin and TSA inhibits SE in IZEs (Wójcikowska et al. 2018). To explore the link between ΔARF5-induced phenotypes and fluctuations in endogenous auxin levels, we examined the expression of auxin biosynthetic genes from the IPyA pathway: *TAA1, TAR1, TAR2*, and the flavin monooxygenases *YUC1-11*. These genes are expressed during the SE process and are critical for auxin production during SE formation (Bai et al. 2013; Li et al. 2022; Karami et al. 2023b). Using the *proXVE:ΔARF5 proDR5:GFP* transgenic line, we induced *ΔARF5* overexpression in explants cultured on 2,4-d supplemented medium with 1 *µ*M β-estradiol for 1, 5, and 10 days. Our analysis revealed that *ΔARF5* overexpression significantly altered the expression of *TAA1*, *TAR1*, *YUC3*, *YUC5*, and *YUC8* genes (Fig. 5A; Supplementary Table S1). Specifically, *TAA1*, *TAR1*, *YUC5*, and *YUC8* showed increased expression levels after 24 h of *ΔARF5* overexpression (Fig. 5A; Supplementary Table S1), whereas *YUC3* and *YUC8* were upregulated on the 5th day (Fig. 5A; Supplementary Table S1). Ten days of *ΔARF5* overexpression maintained the up-regulation of *TAA1* and *YUC5* genes but resulted in the down-regulation of *YUC3* (Fig. 5A; Supplementary Table S1). Further analysis in the *p35S::bdl-GR* line and the *mp* mutant confirmed that *TAA1*, *TAR1*, *YUC3*, *YUC5*, and *YUC8* are strongly down-regulated under conditions of reduced MP function (Fig. 5B and C; Supplementary Table S1). The expression profiles of the other auxin biosynthetic genes in *proXVE:ΔARF5 proDR5:GFP* (Supplementary Fig. S3), *pro35S:bdl-GR,* and *mp* lines (data not presented) were mainly characterized by constant or reduced expression. To understand the biological significance of MP11ir and MP in the *in vivo* regulation of *TAA1*, *TAR1*, *YUC3, YUC5*, and *YUC8* genes, their expression was additionally analyzed in 5- and 10-day-old explants of *proMP:MP11ir*, *proMP:MP,* and *proMP:MP11ir proMP:MP* lines in the *mpS319* background (Fig. 5D and E; Supplementary Table S1). In 5-day-old explants, the expression of all tested genes in the presence of single or combined MP and MP11ir proteins was similar to that in Col-0 (Fig. 5D). In the 10-day-old explants (Fig. 5E), the expression of *TAA1*, *YUC3*, and *YUC5* returned to the level observed in the wild-type genotype Col-0 in the presence of MP11ir, in contrast to *TAR1* and *YUC8*, which remained lower than in Col-0. In the presence of MP alone and in combination with MP11ir, the analyzed genes had the same (*TAA1, YUC8*) or slightly higher (*YUC3*, *YUC5*) expression than in Col-0 and, notably, all genes were characterized by a significantly higher expression than in the *mpS319* mutant. Interestingly, a synergistic effect of MP11ir and MP on the expression of *YUC3* and *YUC8* was significant compared to their individual effect.

Notably, a DAP-seq analysis (O'Malley et al. 2016) identified binding peaks for MP on the promoters of *TAA1*, *TAR1*, and *YUC5* and for ARF2 on the *YUC8* promoter (Supplementary Fig. S4). All the genes deregulated in response to ΔARF5 have at least one ARF-binding peak. These results collectively suggest that MP, ΔARF5, and thus presumably MP11ir, may positively regulate the expression of genes involved in auxin biosynthesis, specifically *TAA1*, *TAR1*, *YUC3*, *YUC5,* and *YUC8* genes.

### The localization of TAA1, TAR1, YUC3, YUC5, and YUC8 enzymes during induction of the SE process is regulated by MP and MP11ir

To elucidate the spatial distribution of the enzymes involved in the IPyA-dependent auxin biosynthesis during the embryogenic transition, we utilized translational reporter lines for TAA1, TAR1, TAR2, and YUCs proteins (Supplementary Fig. S5). The TAA1, TAR1, YUC3, YUC5, and YUC8 enzymes were selected based on the RT-qPCR results (Fig. 5; Supplementary Table S1). To validate the regulation of their expression by MP and ΔARF5, lacking the PB1 domain similar to MP11ir, we crossed the translational reporter lines for TAA1, TAR1, YUC3, YUC5, and YUC8 into *proXVE:ΔARF5 proDR5:GFP, pro35S:bdl-GR*, and *mp* lines to investigate any changes in the localization of GUS signals. Explants were induced into SE with 2,4-d and 1 *µ*M β-estradiol or 10 *µ*M DEX to induce *ΔARF5* or *bdl* overexpression, respectively, and sampled on the 5th and 10th days of the culture. Additionally, *mp* IZE (0 day) and explants on the 5th and 10th days of the SE process were analyzed.

**Figure 6.**
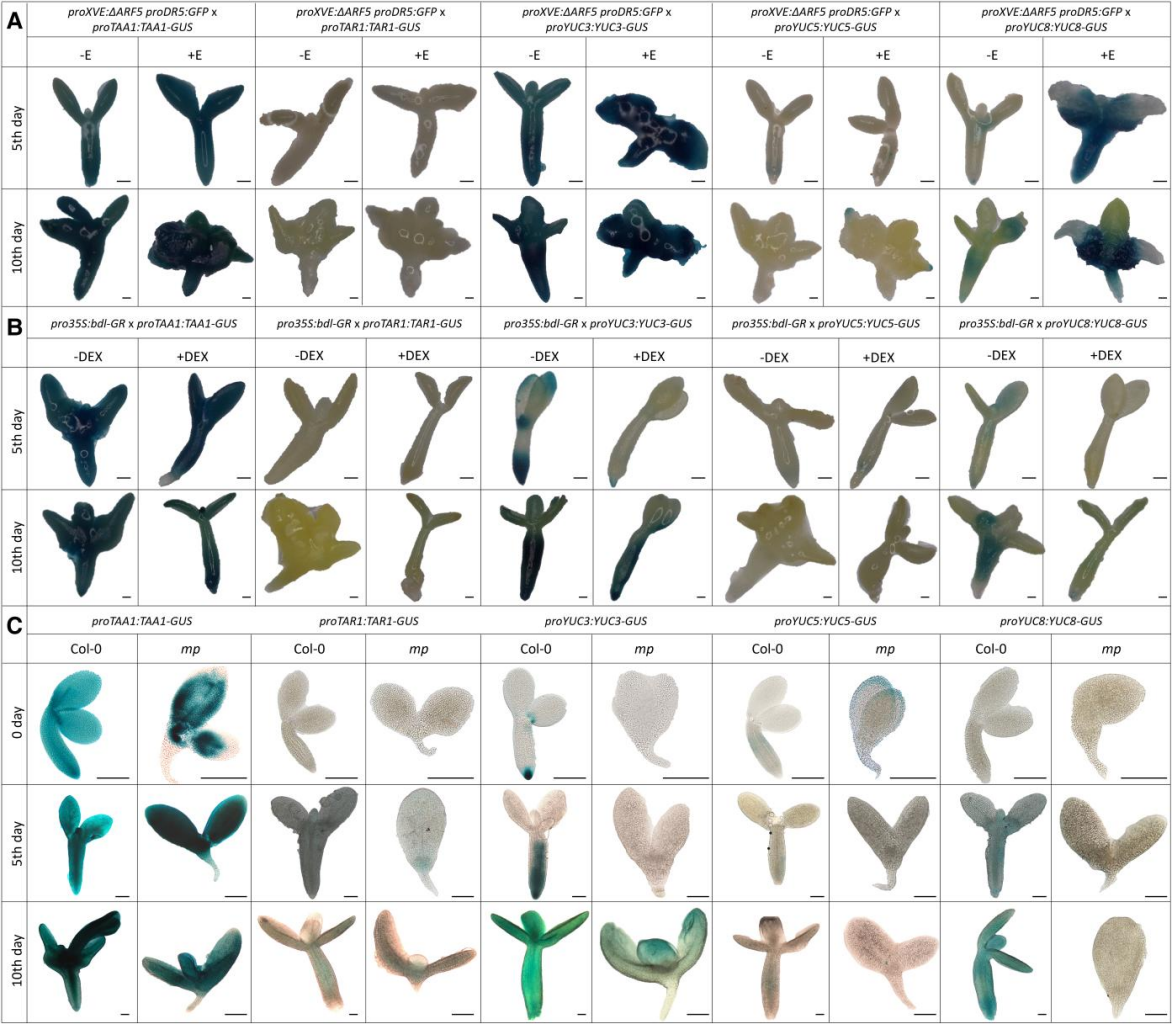
*TAA1*, *TAR1*, *YUC3*, *YUC5*, and *YUC8* expression patterns are altered when MP-dependent transcriptional activities are impaired. Spatio-temporal localization of TAA1, TAR1, YUC3, YUC5, and YUC8 enzymes during SE process under *in vitro* culture in *proXVE:ΔARF5 proDR5:GFP* (**A**) *pro35S:bdl-GR* (**B**) or *mp* background (**C**). IZE explants of reporter lines were cultured on an E5 medium as a control (**A, B, C**), E5 medium with 1 *µ*M β-estradiol for *ΔARF5* overexpression (+E) (**A**), E5 medium with 10 *µ*M dexamethasone (+DEX) (**B**). The tissue was sampled on the 5- and 10-day-old *in vitro* cultures (**A, B**), and in IZEs, explants on the 5- and 10-day-old *in vitro* cultures (**C**). The expression is visible in blue from *proTAA1:TAA1-GUS*, *proTAR1:TAR1-GUS*, *proYUC3:YUC3-GUS*, *proYUC5:YUC5-GUS*, and *proYUC8:YUC8-GUS* reporter lines crossed into the respective genetic backgrounds. Scale bars indicate 200 *μ*m. Images were digitally extracted for comparison. DEX—dexamethasone; IZE—immature zygotic embryo; SE—somatic embryogenesis.

**Figure 7.**
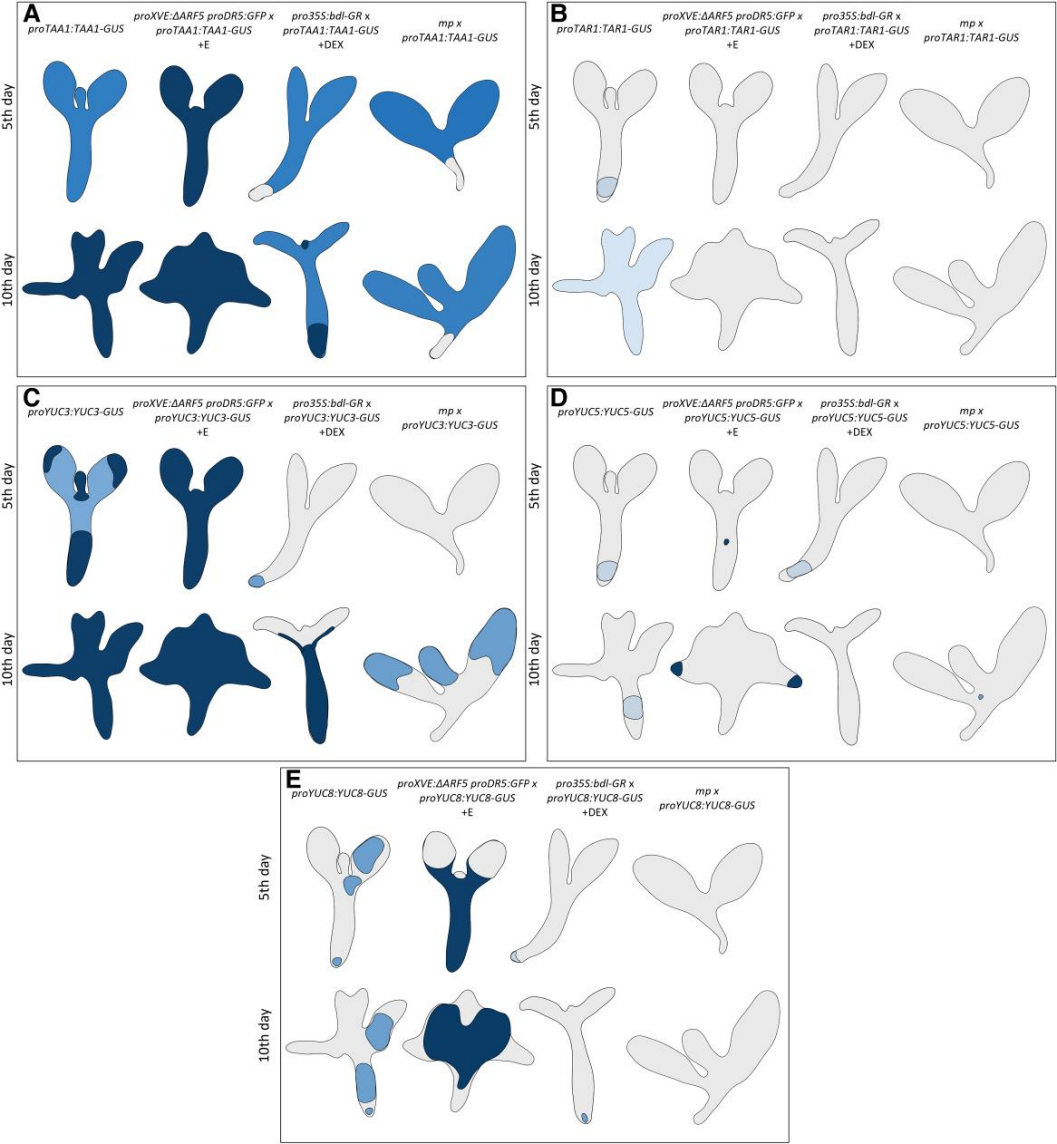
Summary of the localization of TAA1 (**A**), TAR1 (**B**), YUC3 (**C**), YUC5 (**D**), and YUC8 (**E**) enzymes during SE process under *in vitro* culture of Col-0, *proXVE:ΔARF5 proDR5:GFP* line treated with β-estradiol, *pro35S:bdl-GR* line treated with DEX, and *mp* mutant. The figure was created using BioRender. DEX—dexamethasone; SE—somatic embryogenesis.

Among the enzymes analyzed, TAA1, YUC3, YUC5, and YUC8 exhibited distinct expression patterns in response to *ΔARF5* overexpression (Fig. 6). Notably, YUC8 showed robust accumulation in the proximal regions of cotyledons and hypocotyl upon *ΔARF5* overexpression, as evidenced by intense GUS signals (Figs. 6A and 7E). Conversely, *bdl* overexpression led to a significant decrease in GUS signal intensity for YUC8 (Figs. 6B and 7E). In *mp* mutant, the GUS signal for YUC8 was absent (Figs. 6C and 7E), confirming the MP*-* and MP11ir-dependent expression of the *YUC8* gene. TAA1 was broadly active throughout the IZE explants during embryogenic transition (5th and 10th day), with a slight increase of the GUS signal upon *ΔARF5* overexpression (Figs. 6A and 7A). However, changes in GUS intensity were modest upon *bdl* overexpression or in *mp* mutant (Figs. 6B, C and 7A). YUC3 exhibits ubiquitous localization in the hypocotyl region of explants, which is enhanced in the cotyledons upon *ΔARF5* overexpression (Figs. 6A and 7C). In contrast, *bdl* overexpression deeply reduced the intensity of the GUS signal (Figs. 6B and 7C), which was completely absent in *mp* mutant IZEs or reduced at 5th and 10th days of SE induction (Figs. 6C and 7C). YUC5 showed a specific accumulation at the tip of the cotyledons only on the 10th day-old SE under *ΔARF5* overexpression (Figs. 6A and 7D), with no changes observed during *bdl* overexpression (Figs. 6B and 7D). In *mp* mutant, the GUS signal for YUC5 was restricted to single cells (Figs. 6C and 7D). In contrast, TAR1 localization was unchanged upon *ΔARF5,* and *bdl* overexpression, and in *mp* mutant (Figs. 6 and 7B).

These findings demonstrated the cell-type-specific and temporal regulation of the TAA1, YUC3, YUC5, and YUC8 auxin biosynthetic enzymes by ΔARF5, MP, and MP11ir proteins during the SE process.

### Influence of MP11ir on endogenous auxin levels

**Figure 8.**
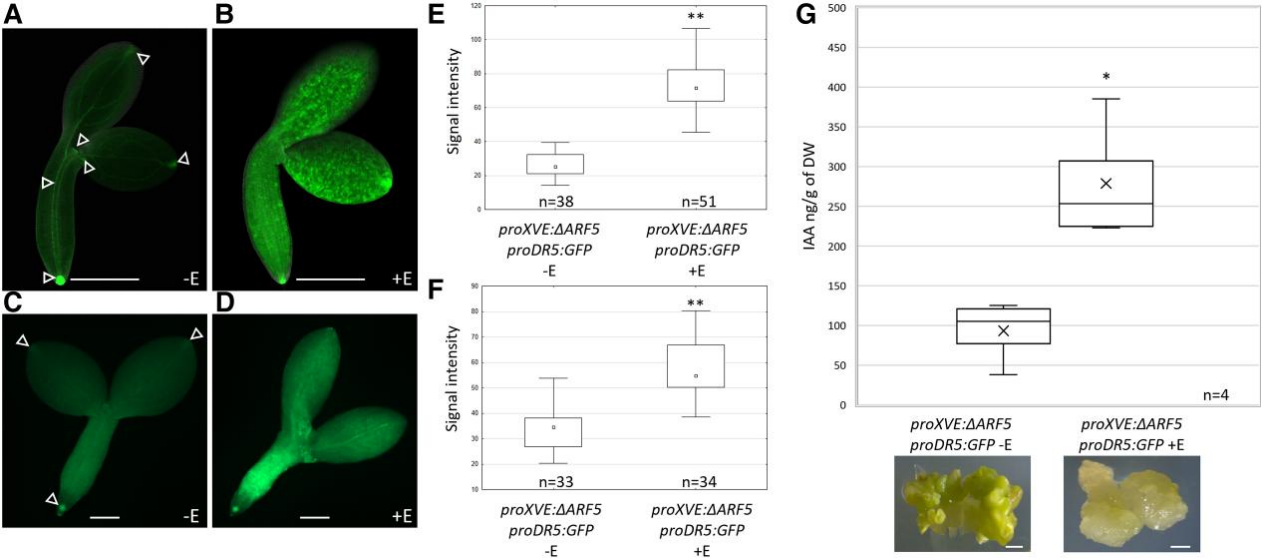
Overexpressing *ΔARF5* increases endogenous auxin levels. **A-D)** GFP-monitored auxin maxima in *XVEpro:ΔARF5 proDR5:GFP* explants cultured for 1 (**A, B**) and 5 days (**C, D**) on an ET medium (1.0 *μ*M TSA) without (−E) (**A, C**) or with β-estradiol (+E) (**B, D**). The GFP fluorescence signal is displayed in green. **E, F)** The fluorescence signal was quantified in 1- (**E**) and 5-day-old (**F**) explants. The data are presented as a boxplot. The cross represents the mean value; the center line represents the median value; the box limits are upper and lower quartiles; the whiskers show the 1.5 × interquartile range. **G)** Level of IAA (ng/g of dry weight DW) in 10-day-old IZE-culture of *proXVE:ΔARF5 proDR5:GFP* with 1 *µ*M β-estradiol to induce *ΔARF5* overexpression. A Student *t*-test was used to determine any values that were significantly different (*P < 0.05; **P < 0.01). The images are identical to those used in Fig. 4C. They illustrate the type of analyzed samples. Scale bars indicate 200 *μ*m (**A-D**) and 1 mm (**G**). Arrowhead (**A, C**) marked regions of high auxin response in *proXVE:ΔARF5 proDR5:GFP* explants untreated by β-estradiol (−E). E—β-estradiol; IAA—indole-3-acetic acid; IZE—immature zygotic embryo; TSA—trichostatin A.

We hypothesized that MP11ir might regulate the endogenous auxin levels through TAA1, YUC3, YUC5, and YUC8 during SE. We explored this hypothesis using three approaches. First, we assessed local auxin responses in the *proXVE:ΔARF5 proDR5:GFP* explants during the auxin-independent embryogenic transition (1st and 5th days of TSA treatment) to avoid activation of the *DR5* signal by the exogenously applied auxin. Explants were cultured on an ET medium with or without β-estradiol. GFP signals indicate auxin-responsive gene transcription in the root and SAMs and the tip of cotyledons in non-induced explants (Fig. 8A). After 24 h of induced *ΔARF5* overexpression, an enhanced GFP signal was observed in the hypocotyl and throughout the cotyledons (Fig. 8B and E). In 5-day-old explants, *ΔARF5* overexpression intensified the GFP signal in the hypocotyl and cotyledons (Fig. 8C, D, and F). Second, we quantified indolic compounds and IAA in 21- or 10-day-old *in vitro* cultures, respectively, of *proXVE:ΔARF5 proDR5:GFP* explants treated and not with β-estradiol (Supplementary Fig. S6; Fig. 8G). The explants overexpressing *ΔARF5*, exhibiting callus formation, showed a 1.37-fold increase in indolic compound levels and a 3-fold increase in IAA content compared to non-induced explants. Finally, to determine whether the formation of calli might result from high auxin responses and indolic compound levels, we analyzed the SE process in Col-0 and the *proXVE:ΔARF5 proDR5:GFP* line treated with different concentrations of yucasin (100 and 150 *µ*M), an inhibitor of YUC activity (Tsugafune et al. 2017). (Non-)induced Col-0 (−E, +E) and non-induced *proXVE:ΔARF5 proDR5:GFP* (−E) explants treated with yucasin showed highly reduced SE efficiency (1.3 to 1.5 times lower) and productivity (no more than two somatic embryos per reacting explant were formed) (Supplementary Fig. S7). The results align with published literature (Li et al. 2022; Karami et al. 2023b). We then investigated whether treating *proXVE:ΔARF5 proDR5:GFP* IZE explants with yucasin could reverse the phenotype induced by *ΔARF5* overexpression with β-estradiol and enable the explants to regenerate embryos instead of callus tissue. Indeed, these *proXVE:ΔARF5 proDR5:GFP* IZE explants formed a few somatic embryos (15.4% with 100 and 3.4% with 150 *µ*M yucasin) and with low productivity (1 somatic embryo per reacting explant) instead of the calli observed in the absence of yucasin (Supplementary Fig. S7).

This finding supports that *ΔARF5* overexpression amplifies local auxin responses by modulating auxin production and signaling during SE, ultimately forming calli instead of embryonic structures. However, other genetic components deregulated by *ΔARF5* overexpression also likely inhibit SE capacity.

## Discussion

*MP* is upregulated in plant embryogenic cells and serves as a potential molecular marker for this developmental stage. Various transcriptomic studies support the involvement of MP in the SE process in various plant species (Thibaud-Nissen et al. 2003; Gliwicka et al. 2013; Wickramasuriya and Dunwell 2015; Indoliya et al. 2016; Shi et al. 2016; Capote et al. 2019; Juárez-González et al. 2019; Quintana-Escobar et al. 2019; Chen et al. 2020; Qi et al. 2021; Awada et al. 2023; Zhu et al. 2023; Xu et al. 2024; Zeng et al. 2024; Ge et al. 2025). Additionally, the presence of *MP* transcripts in regions critical for SE induction, such as cotyledons and SAM, suggests that MP may be involved in the embryogenic transition (Wójcikowska and Gaj 2017). Two *mp* mutant alleles, *arf5* (SALK_001058C) (Wójcikowska and Gaj 2017) and *mpS319* (SALK_021319) (present work), exhibit impaired SE processes. This inhibition is linked to the differential expression of SE master regulators (Wójcikowska and Gaj 2017). Similarly, the *Osarf5* mutant displays partial defects in forming a scutellum-derived embryogenic callus in rice. The *Osarf5* mutant also shows a significantly reduced expression of *LEAFY COTELYDON 1* (*OsLEC1*), indicating that the OsARF5-mediated auxin signaling pathway might regulate *OsLEC1* (Guo et al. 2023a). Overexpression of *MdARF5* induced the SE process in apple, which shortened the induction cycle and improved the efficiency of the SE process (Yang et al. 2024b).

Expressing auxin-insensitive bdl protein during SE induction revealed that SE inhibition in the *mp* mutant is not due to its embryonic defects but is triggered by MP inactivity in IZE. Many *EMBRYO-DEFECTIVE* (*EMB*) genes involved in zygotic embryo development and morphogenesis, such as *LEC1*, *LEC2*, *FUSCA 3*, *DICER-LIKE 1*, *WUSCHEL RELATED HOMEOBOX 5* (*WOX5*), and *PIN-FORMED 1* (*PIN1*), are also critical for SE processes (Ledwoń and Gaj 2009; Su et al. 2009; Ledwoń and Gaj 2011; Su et al. 2015; Szyrajew et al. 2017; Meinke 2020). However, the overexpression of the auxin-insensitive bdl protein had a more substantial impact on inhibiting embryogenic transition than the *mp* mutation, suggesting that the mutated bdl protein might repress other ARFs besides MP (Piya et al. 2014).

MP's transcriptional targets likely include genes encoding proteins essential to the SE process. AGAMOUS-LIKE 15 (AGL15) and AGL18, crucial for SE (Harding et al. 2003; Thakare et al. 2008; Zheng and Perry 2014; Paul et al. 2022), might regulate *MP* expression as the *agl15 agl18* double mutant shows a significant reduction of *MP* mRNA levels (Zheng et al. 2009). Also, inhibition of PHYTOCHROME-INTERACTING FACTOR 4 (PIF4) induces several auxin responses (*ARF5, ARF8,* and *ARF16*) and biosynthetic (*YUC1, YUC2*, *YUC6*, *CYP79B2,* and *AMIDASE 1* (*AMI1*)) genes, resulting in the formation of the somatic embryos (Mira et al. 2023).

Despite these insights, the detailed mechanism of action of MP and its MP11ir isoform during SE induction and somatic embryo formation remains unknown. Lines expressing truncated MP proteins without the PB1 domain could elucidate the complex action of MP11ir by mimicking its function. A semi-dominant, gain-of-function allele of *MP* is encoded by the truncated MP^abn^ protein. Both ΔARF5 and MP^abn^ proteins partially rescue *mp* phenotypic defects but cause unique leaf defects (Garrett et al. 2012; Krogan et al. 2012). Ectopic ΔARF5 protein stimulates the formation of new shoots *in vivo* (Ma et al. 2019), whereas *mpS319* mutants show reduced shoot regeneration frequency (Zhang et al. 2021). During *in vitro* culture, ΔARF5 increases the shoot organogenesis frequencies in Arabidopsis root-derived explants and activates auxin signaling. Visualization and cytometric quantification of the *proDR5:GFP* nuclear auxin signaling reporter revealed an increased GFP signal in roots and protoplasts overexpressing *ΔARF5* (Gonzalez et al. 2021). A similar trend was observed after inducing *ΔARF5* expression during the embryogenic transition. MP is involved in SAM activity and maintenance by directly controlling the expression of *ARABIDOPSIS HISTIDINE PHOSPHOTRANSFER PROTEIN 6* (*AHP6*) (Besnard et al. 2014), *TARGET OF MONOPTEROS* (*TMOs*) genes (Schlereth et al. 2010), *DORNRÖSCHEN/ENHANCER OF SHOOT REGENERATION 1* (*DRN/ESR1*) (Cole et al. 2009), *WUSCHEL RELATED HOMEOBOXs* (*WOXs*) (Tvorogova et al. 2019; Lee et al. 2022), essential for shoot regeneration or SE (Fambrini et al. 2022). The shoot regeneration-promoting effect of ΔARF5 (Ckurshumova and Berleth 2015; Gonzalez et al. 2021) may help to overcome organogenic resistance in recalcitrant explants and species in the future. However, *ΔARF5* overexpression did not enhance embryogenic capacity (our results) but instead led to callus formation, similar to *NcARF5* overexpression in kadamba (*Neolamarckia cadamba*), which triggers rapid callus proliferation (Ma et al. 2024).

Studies on embryo-derived SE systems showed that SEs initiate from the callus edge, where auxin accumulates, as visualized using PIN1 and DR5 reporters (Su et al. 2009; Su et al. 2015; Kadokura et al. 2018). MP directly and positively regulates *PIN1*, which is responsible for auxin efflux in polar auxin transport (PAT) (Krogan et al. 2016). Auxin is present in the *pin1* mutant meristem, ensured by vascular tissue transport or induction of auxin biosynthesis (Banasiak et al. 2019). Therefore, MP is proposed as a primary regulator of auxin transport and biosynthesis. Our study identifies auxin biosynthetic genes, *TAA1*, *TAR1*, *YUC3*, *YUC5,* and *YUC8*, as potentially positively regulated by ΔARF5, MP11ir, and/or MP during *in vitro* embryogenic transition. We propose that MP and MP11ir proteins directly or indirectly control the TAA1/TARs-YUCs-mediated auxin biosynthetic pathway (our work) and polar auxin transport during embryogenesis (Schlereth et al. 2010; Robert et al. 2015). This hypothesis aligns with findings that MP affects *YUC1* and *YUC8* expression to specify the ground tissue in early globular zygotic embryos (Möller et al. 2017). IPyA-dependent auxin biosynthesis is crucial for effective SE induction and somatic embryo identity and growth (Li et al. 2022). Yucasin treatment, inhibiting YUC activity (Tsugafune et al. 2017), reduces SE efficiency and productivity in Arabidopsis (Li et al. 2022; Karami et al., 2023b) and coffee culture (Uc-Chuc et al. 2020). The importance of auxin biosynthesis in the SE process is evidenced by the decreased embryogenic response in *yuc* mutants. Multiple *yuc* mutants, *yuc3 yuc8* (Li et al. 2022), *yuc1 yuc4 yuc10 yuc11* (Bai et al. 2013), *yuc3 yuc5 yuc7 yuc8 yuc9* (Sakamoto et al. 2022), display disrupted SE or callus formation compared to single mutants (Wójcikowska et al. 2013; Sakamoto et al. 2022). *YUC* genes are direct targets of TFs, such as LEC1, LEC2, BABY BOOM (BBM), and AT-HOOK MOTIF CONTAINING NUCLEAR LOCALIZED 15 (AHL15), which play pivotal roles in embryogenic transition, demonstrated by decreased SE in their mutant and spontaneous induction of somatic embryo formation when overexpressed (Lotan et al. 1998; Stone et al. 2001; Boutilier et al. 2002; Gaj et al. 2005; Ledwoń and Gaj 2009; Karami et al. 2021). During SE induction, LEC1 activates *YUC4* and *YUC10* (Junker et al. 2012; Hu et al. 2018), while LEC2 positively regulates *YUC1*, *YUC4*, and *YUC10* expression (Stone et al. 2008; Wójcikowska et al. 2013). BBM induces local and ectopic expression of *YUC3* and *YUC8* coupled with IAA biosynthesis and activation of the WOX2 embryo marker (Li et al. 2022). During SE induction, AHL15 activates *YUC6*, *YUC7*, *YUC8*, and *YUC9* expression (Karami et al. 2023b), confirming YUCs as crucial molecular elements of a network governing the SE process.

We showed that both *MP11ir* and *MP* transcript levels increase along with the concentration of auxins (2,4-d, NAA, IAA). The auxins analyzed in our study exhibited different potencies in inducing SE, and with increasing auxin concentrations, a greater ability to induce SE was observed. Similarly, the synthetic auxin 2,4-d was effective in SE induction, whereas natural auxins were less efficient or even failed to induce regeneration (Karami et al. 2024). Why do auxins act differently? 2,4-d cannot be efficiently transported and accumulates in the cells, triggering a strong (optimal for SE) auxin response. In contrast, natural auxins are subject to polar transport, which prevents their local accumulation and results in a moderate auxin response—insufficient for SE induction. It has been shown that both natural auxins and synthetic analogs become efficient inducers of SE when their efflux is transiently inhibited by an auxin transport inhibitor. Explants of auxin efflux mutants, such as *pin2* and *abcb1 abcb19*, show enhanced SE efficiency when treated with IAA or efflux-inhibited IAA, confirming that auxin efflux reduces the efficiency of SE in Arabidopsis. Efflux-inhibited IAA, similar to 2,4-d, also efficiently induced SE from carrot suspension cells, and efflux-inhibited 4-Cl-IAA improved de novo shoot regeneration in oilseed rape (*Brassica napus*) (Karami et al. 2024).

A study identified target genes of MP and truncated ΔARF5 proteins in 3-day-old seedlings (un)treated with auxin (Xie et al. 2022). This ChIP-seq analysis suggests that deleting the PB1 domain renders the protein auxin-insensitive and alters its binding profile. Among genes involved in the IPyA auxin biosynthesis pathway, *YUC3* and *YUC5* promoters are recognized and bound by ΔARF5, while the *YUC6* promoter is targeted by ΔARF5 and MP. Differences in the preferential AuxRE motifs bound by ΔARF5 and MP suggest that the PB1 domain may affect homodimer formation and DNA binding, indicating that ΔARF5 and MP may differ in their molecular actions and thus impact plant development differently (Xie et al. 2022).

Similarly, recent transcriptomic data revealed that MP and MP11ir may regulate the expression of different sets of genes during Arabidopsis root development and have specific and non-redundant functions. Among DEGs, *TAR2* (1.6 and 1.7-fold) and *YUC9* (1.5- and 1.8-fold) can be distinguished with an increased expression in *proMP:MP* or *proMP:MP11ir* roots, respectively, relative to the wild type. Other genes involved in Trp (IAA precursor) (*IGPS*, *TRP1*, *TSA1*, *TSBtype2*) and IAA biosynthesis (*AMI1*, *NIT1*-3, *IAMT1*, *CYP79B3*) or conjugation (*GH3.2/BRU6/AUR3, GH3.6/DFL1, GH3.18, MES17*) are also deregulated in an MP or MP11ir-dependent manner in root cells (Cavalleri et al. 2024). Also, during zygotic embryogenesis, the expression of *GRETCHEN HAGEN GH3.1* and *GH3.6* genes seems to be MP-dependent (Schlereth et al. 2010). MP, like other ARF proteins (Takase et al. 2004; Tian et al. 2004; Mallory et al. 2005), may play a role in regulating the conversion of IAA into storage forms, involving GH3 proteins—acyl-amido synthetases mediate the conjugation of IAA with amino acids (Staswick et al. 2005). It suggests that MP and MP11ir may regulate the expression of genes involved in auxin metabolism and modulate the cell's responsiveness to auxin.

Surprisingly, the lack of a GFP signal in the *proMP:MP11ir-GFP* SE makes it impossible to follow the isoform localization. This may be due to a weak GFP signal, below the detection threshold, or to post-transcriptional or post-translational modifications of the MP11ir-GFP proteins. The *proMP:MP-EGFP(MR)* line, in which the EGFP sequence is inserted into the MR domain of MP, upstream of the differentially spliced 11th intron, was used to localize MP11ir in root (Cavalleri et al. 2024). Such a line, together with the *proMP:MP-VENUS-2Ap-mTURQUOISE,* may allow the localization of MP and MP11ir proteins during the SE process.

Transcriptomic data have shown that many splice isoforms formed due to AS show cell-specific expression (Klepikova et al. 2016; Martín et al. 2021). Among AS events, intron retention events are functionally underestimated in plants. Given the importance of AS in responding to various stresses, it is likely required for proper environmental response in plants (Guo et al. 2023b). Limited functional evidence supports their specific expression patterns or distinct ability to rescue mutant loss-of-function phenotypes. For example, *ARF8* undergoes tissue-specific AS in the flower, where the *ARF8*.*4* variant, which retains the eighth intron and has a premature stop codon, is over-represented. Overexpression of *ARF8.4*, but not other *ARF8* isoforms, reverts stamen elongation defects associated with the *arf8* knockout mutation (Ghelli et al. 2018; Ghelli et al. 2023). Another example is the *BIGPETAL* (*BPE*) gene, which encodes a petal-specific *BPEp* transcript that retains its last intron, in addition to the canonical *BPEub* transcript uniformly expressed in all organs (Szécsi et al. 2006). The C-terminal sequence exclusively encoded by *BPEp* is required to interact with ARF8. The *arf8* loss-of-function mutant phenocopies the petal defects of the *bpe* knockout mutants, suggesting that BPEp, unlike the canonical BPEub protein, is required for petal development (Varaud et al. 2011; Kashkan et al. 2022).

**Figure 9.**
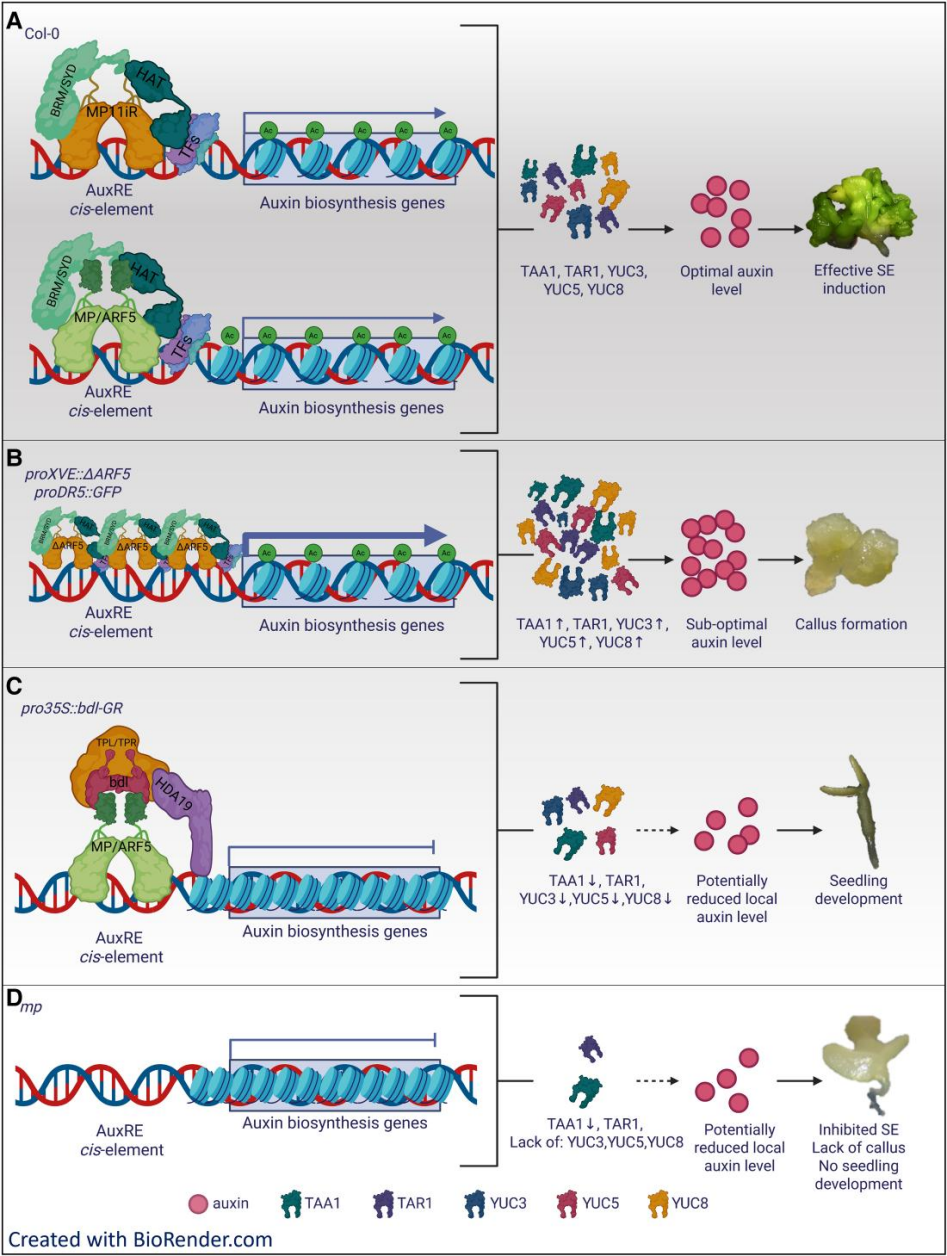
Proposed model of MP and MP11ir action during embryogenic transition through control of auxin-biosynthesis genes expression. **A)** The canonical MP protein and MP11ir isoform are required for the efficient embryogenic transition by controlling auxin biosynthesis genes. **B)** Overexpression of a truncated *ΔARF5* protein caused elevated levels of TAA1, YUC3, YUC5, and YUC8 enzymes in explants, which disrupts auxin homeostasis, leads to suboptimal endogenous auxin levels, inhibits the SE process, and induces the formation of callus. The image to illustrate the formation of a callus has been reused from Fig. 4C, bottom row. **C)** Blocking MP action through the expression of the *bdl* repressor results in decreased TAA1, YUC3, YUC5 and YUC8 levels during SE induction, inhibits the SE process, and induces seedling development. **D)** The lack of MP and MP11ir in the *mp* mutant leads to decreased TAA1 level, absence of YUC3, YUC5, and YUC8 enzymes during the early stage of SE induction, which inhibits the SE process, and leads to callus formation. The image to illustrate the inhibition of SE and formation of a callus has been reused from Fig. 2J. AuxRE—Auxin Response Elements; BRM/SYD—BRAHMA/SPLAYED; MP—MONOPTEROS; MP11ir—MP isoform; TFs—TRANSCRIPTION FACTOR; HAT—HISTONE ACETYLASES; TAA1—TRYPTOPHAN AMINOTRANSFERASE OF ARABIDOPSIS 1, TAR1—TRYPTOPHAN AMINOTRANSFERASE RELATED 1; TPL/TPR—TOPLESS/TPL-RELATED; YUC—YUCCA; Ac—acetyl group; SE—somatic embryogenesis. Created with BioRender.com.

In conclusion, both MP proteins, canonical MP and MP11ir isoform, seem to be involved in the SE process (Fig. 9A). Potentially different expression patterns of MP and MP11ir suggest that they act in different cells, probably with different endogenous auxin levels. The ectopic expression of *ΔARF5,* results in a global increase in endogenous auxin content in explants through direct or indirect activation of *TAA1*, *TAR1*, *YUC3*, *YUC5,* and *YUC8* genes. Disruption of auxin homeostasis in explant cells by overproduction of TAA1, YUC3, YUC5, and YUC8 enzymes leads to SE inhibition and callus formation (Fig. 9B). Blocking MP action by expressing the *bdl* repressor results in decreased levels of TAA1, YUC3, YUC5 and YUC8 enzymes during SE induction, inhibiting the SE process and promoting seedling development (Fig. 9C). The absence of both MP and MP11ir in *mp* mutant leads to the decreased TAA1 level, absence of *YUC3*, *YUC5,* and *YUC8* during early stage of SE induction, resulting in SE inhibition and callus formation (Fig. 9D). However, the analysis of auxin biosynthetic gene expression in the complemented lines, expressing MP (*proMP:MP mpS319*), MP11ir (*proMP:MP11ir mpS319*) or both proteins together (*proMP:MP proMP:MP11ir mpS319*), suggest that MP and MP11ir may have common and specific transcriptional targets during SE, with MP11ir regulating *TAA1*, *YUC3*, and *YUC5*, MP regulating *YUC8* and a synergistic MP and MP11ir effect on *YUC3* and *YUC5*. In conclusion, by suggesting that MP and MP11ir are central regulators of auxin biosynthesis during embryogenic processes, this study provides an elegant and straightforward experimental setup that elucidates the role of MP and MP11ir in SE induction, acting through the transcriptional control of auxin biosynthetic genes.

## Materials and methods

### Plant material

The seeds of Arabidopsis (*Arabidopsis thaliana* (L.) Heynh) Columbia (Col-0) wild type, *mpS319* mutant (N21319), reporter lines *proTAA1:TAA1-GUS* (N72246), *proTAR1:TAR1-GUS* (N72247), *proTAR2:TAR2-GUS* (N72248), *proYUC1:YUC1-GUS* (N72250), *proYUC2:YUC2-GUS* (N72252), *proYUC3:YUC3-GUS* (N72254), *proYUC4:YUC4-GUS* (N72256), *proYUC5:YUC5-GUS* (N72259), *proYUC6:YUC6-GUS* (N72261*), proYUC7:YUC7-GUS* (N72262), *proYUC8:YUC8-GUS* (N72264), *proYUC9:YUC9-GUS* (N72266), *proYUC10:YUC10-GUS* (N72268), and *proYUC11::YUC11-GUS* (N72271) were supplied by NASC (The Nottingham Arabidopsis Stock Centre) (Alonso et al. 2003; Brumos et al. 2020). Seeds of the *proMP:MP mpS319, proMP:MP11ir-GFP mpS319*, and *proMP:MP11ir-GFP* transgenic lines were provided by Lucia Colombo (Dipartimento di BioScienze, Università degli Studi di Milano, Milano, Italy). Dolf Weijers (Laboratory of Biochemistry, Wageningen University, and Research, Wageningen, The Netherlands) generously donated seeds of the *proMP:MP-GFP*, *pro35S:BDL-GR*, *pro35S:bdl-GR* lines. The seeds of the *proXVE:ΔARF5 proDR5:GFP* line were provided by Bastiaan Bargmann (School of Plant and Environmental Sciences, Virginia Polytechnic Institute and State University, Blacksburg, USA). The generation of *proMP:MP mpS319×proMP:MP11ir-GFP mpS319*, and *proXVE:ΔARF5 proDR5:GFP*; *pro35S:bdl-GR; arf5×proTAA1:TAA1-GUS*, *proTAR1:TAR1-GUS*, *proYUC3:YUC3-GUS*, *proYUC5:YUC5-GUS*, *proYUC8:YUC8-GUS* was performed by crossing.

### Plant growth and *in vitro* culture conditions

Seeds were sterilized with sodium chloride (20% commercial bleach) and plated onto ½ MS medium (Murashige and Skoog 1962), (Duchefa Biochemie; #M0222), 10 g L^−1^ sucrose (Penta #57-50-1) and 8 g L^−1^ agar (Duchefa Biochemie; #P1001). Seed-derived plants grown *in vitro* were a source of seedlings, and *in vivo* were a source of leaves, flowers, and siliques for IZE isolation. Plants were grown in soil with vermiculite (4:1) at 22 °C under a 16 h photoperiod of 100 *µ*M m^−2^ s^−1^ white, fluorescent light.

### Somatic embryogenesis induction

IZEs at the cotyledonary stage of development were used as explants for the *in vitro* culture. The IZEs were cultured on an E5 medium containing 3.2 g L^−1^ of B5 micro and macro-elements (Duchefa Biochemie; #G0210) (Gamborg et al. 1968), 20 g L^−1^ sucrose, 5.0 *μ*M of 2,4-d (2,4-dichloro phenoxy acetic acid, Sigma-Aldrich #D7299), and 8 g L^−l^ agar (Duchefa Biochemie; #P1001), pH 5.8 (Gaj 2001). To induce the SE process without auxin, the IZEs were cultured on an ET medium containing 3.2 g L^−1^ of B5 micro and macro-elements (Duchefa Biochemie; #G0210) (Gamborg et al. 1968), 20 g L^−1^ sucrose, 1 *μ*M of TSA (Sigma Aldrich; #T1952) and 8 g L^−l^ agar (Duchefa Biochemie; #P1001), pH 5.8 (Wójcikowska et al. 2018). Plant materials that grow in sterile conditions were kept at 23 °C under a 16 h photoperiod of 40 *µ*M m^−2^ s^−1^ white, fluorescent light. The explant capacity for SE was evaluated in an IZE-derived culture that was induced for 21 days on an induction media, and two parameters were calculated—SE efficiency (percentage of explants that developed somatic embryos) and productivity (average number of somatic embryos produced by embryogenic explant). Thirty explants were analyzed in at least three biological replicates of the experimental combination. Around 10,000 IZEs were isolated for all the experiments. Assuming the average embryo isolation rate (100 IZEs per hour), the time to obtain the research material for each experiment was 100 h.

### IZE treatments

IZEs at the cotyledonary stage were treated with different 2,4-d (Duchefa Biochemie; #D0911), IAA (Duchefa Biochemie; #I0901) and NAA (Duchefa Biochemie; #N0903) (0.0, 2.5, 5.0, 7.5 *μ*M) concentrations for ten days to evaluate the level of *MP* and *MP11ir* transcripts. IZEs were cultured on a medium with 2,4-d and treated with different yucasin (Merck; #23711-26-4) concentrations (0, 100, 150 *μ*M) for 3 weeks to evaluate the effect of YUC activity inhibition on the embryogenic capacity. Explants were first precultured for 1 day on a medium without yucasin and transferred to a medium with different yucasin concentrations to allow for IZE survival. Treating IZE explants with yucasin from the beginning of the culture (without preculture) resulted in the death of the explants (Karami et al. 2023b).

### Induction of *BDL*, *bdl*, and *ΔARF5* expression in transgenic plants

The action of BDL and bdl was controlled by adding dexamethasone (water-soluble DEX, Sigma; #D2915) to the media at a concentration of 10 or 30 *µ*M. The expression of *ΔARF5* was controlled by adding 1 *µ*M β-estradiol (ethanol-soluble E, Sigma; #E8875) to the media.

### RNA isolation, reverse transcription, qualitative and quantitative PCR

RNA was isolated from different samples, depending on culture age, tissue from 300 (0, 1st day), 150 (5th day) to 50 (10th day) explants were used for RNA extraction. A NucleoSpin RNA Plant Kit (Macherey-Nagel) was used to isolate the RNA from the samples. The RNAs were treated with RQ1 RNase-free DNase I (Promega) to control DNA contamination according to the manufacturer's instructions. The first-strand cDNA was produced from 1,000 ng of RNA using a RevertAid First Strand cDNA Synthesis Kit (Fermentas). The product of the reverse transcription was used for RT-PCR and RT-qPCRs to detect and quantify gene transcripts. Undiluted cDNA (1 *µ*L) was used for RT-PCR. cDNA diluted with water at a 1:4 ratio (2.5 *µ*L) was used for the RT-qPCRs. RT-qPCR was carried out in a 10 *µ*L reaction volume using a LightCycler 480 SYBR^TM^ Green I Master (Roche) kit, a LightCycler 96 Multiwell Plate 96, and Multiwell Sealing Foil (Roche). The relative RNA levels were calculated and normalized to the internal control of the AT4G27090 (*TIN*) gene encoding the 60S ribosomal protein (Thellin et al. 1999). Fold change values were calculated using the comparative 2^−ΔΔCt^ method, where ΔΔCt represents Δ*Ct*^reference condition^ − Δ*Ct*^compared condition^. The plant tissues for gene expression analysis were produced in three biological replicates, and two technical replicates were analyzed. Primer sequences for *MP11ir*, *MP*, *TIN*, *TAA1*, *TARs*, and *YUCCAs* are in Supplementary Table S2.

### GUS staining

The GUS enzyme activity was performed as described by Bassuner et al. (2006). The explants were stained in a standard X-Gluc solution at 37 °C for 12 h. The experiments were repeated two times for each studied combination, and at least 30 somatic embryos were subjected to GUS signal detection in each repetition. The GUS staining pattern was analyzed using a microscope Zeiss Axioscope.A1 ZEN.

### ClearSee alpha clearing and Renaissance SR2200 staining

The explants were fixed using a fixative solution (4% paraformaldehyde in 1 × phosphate-buffered saline (PBS) with 0.05% Triton-X100 (PBS-T)) and incubated in a 1.5 mL fixative in a microcentrifuge tube for 1 h at room temperature. The tubes were covered with aluminum foil throughout the procedure to be light-tight. The samples were vacuum infiltrated for ∼30 min on ice to remove air bubbles. Then, the samples were incubated overnight at 4 °C with slow rotation. The following day, samples were washed three times with 1 × PBS-T at room temperature for 1 h each. A ClearSee alpha solution was prepared according to Kurihara et al. (2021). The ClearSee solution was supplemented with 50 mm sodium sulfite as an antioxidant. The clearing was extended to 7 days with gentle rotation (10–20 rpm) at room temperature to ensure thorough tissue penetration and clearing. After clearing, a staining step was performed by mixing a 0.1% (v/v) Renaissance SR2200 (Renaissance Chemicals, UK) solution in a ClearSee alpha solution (Attuluri et al. 2022). After staining, the samples were washed in a fresh ClearSee alpha solution for 1 h and mounted on slides.

### Microscopy

Expression pattern analysis was performed on a ZEISS AxioZoom.V16 microscope equipped with an Apotome2 module, allowing for an overview of GFP signal localization. For higher resolution and subcellular localization, particularly to assess nuclear localization of the GFP signal, samples were subsequently examined using a ZEISS LSM 800 laser scanning confocal microscope with a correction collar 10 × magnification objective at 1,024 × 1,024 pixel resolution. Two laser lines were used: 405 nm for imaging Renaissance, combined with 488 nm for GFP imaging. The images were captured in 12 bits. The laser intensity and detector gain were kept constant for all samples.

### Evaluation of indolic compound content

A colorimetric technique that enabled the detection of indolic compounds, including IAA, was applied (Bric et al. 1991; Wójcikowska et al. 2013). Each analysis was carried out in five biological replicates.

### Evaluation of IAA content

The concentration of indole-3-acetic acid (IAA) was evaluated in a 10-day-old IZE culture of *proXVE:ΔARF5 proDR5:GFP* transgenic line with 1 *µ*M β-estradiol-induced *ΔARF5* overexpression as described by Li-Marchetti et al. (2015). Fresh tissue was immediately frozen in liquid nitrogen and stored at −80 °C. The samples were freeze-dried and ground. Approximately 3 mg of dry powder was extracted for each sample with 0.8 mL of acetone/water/acetic acid (80/19/1 v:v:v). Indole-3-acetic acid stable labeled isotope used as an internal standard was prepared, as described by Le Roux et al. (2014). Analysis was performed on four biological replicates.

### ARF binding regions

We employed the DAP-Seq profiles for the MP and ARF2 TFs (O'Malley et al. 2016) available at the PlantCistrome database (http://neomorph.salk.edu/PlantCistromeDB). We analyzed (−−1500; +100) *TAA1/TAR* and *YUC* gene promoters and detected TGTCnn sequences and ARF-binding DAP-Seq peaks. We utilized a suite of R packages, including biomaRt, dplyr, and ggplot2, for computational analysis and data visualization.

### Statistical analysis

Phenotype scoring and quantification data were collected in Excel (Microsoft Corp.) and imported into Statistica (TIBCO Software Inc., Palo Alto, CA, USA) for statistical analysis and to generate graphs. For one-on-one comparisons, the Student's *t*-test was used (*P* < 0.05). For intercomparison of more than two data points, the two-way analysis of variance analysis (*P* < 0.05) followed by Tukey's honest significant difference test (*P* < 0.05, and *P* < 0.01) was used to determine any significant differences between the compared combinations. The graphs show the means with the standard deviation.

### Accession numbers

ARF5/MP AT1G19850; IAA12/BDL AT1G04550; TAA1 AT1G70560; TAR1 AT1G23320; TAR2 AT4G24670; YUC1 AT4G32540; YUC2 AT4G13260; YUC3 AT1G04610; YUC4 AT5G11320; YUC5 AT5G43890; YUC6 AT5G25620; YUC7 AT2G33230; YUC8 AT4G28270; YUC9 AT1G04180; YUC10 AT1G48910; YUC11 AT1G21430; TIN AT4G27090.

## Supplementary Material

kiaf602_Supplementary_Data

## Data Availability

Raw images and source data are provided in the Zenodo repository (https://doi.org/10.5281/zenodo.15357086).

## Supplementary data

The following materials are available in the online version of this article.

**Supplementary Figure S1.**  *MP11ir* is expressed during somatic embryogenesis.

**Supplementary Figure S2.** The induction of somatic embryogenesis depends on 2,4-d and NAA, but not IAA.

**Supplementary Figure S3.** Expression level of auxin biosynthetic genes in *proXVE:ΔARF5 proDR5:GFP* somatic embryos.

**Supplementary Figure S4.** Analysis of the *TAA1*, *TAR1*, and *YUC* promoters for MP binding sites.

**Supplementary Figure S5.** Expression pattern of TAA1, TAR1, TAR2, and YUC1-11 during somatic embryogenesis induction.

**Supplementary Figure S6.** Overexpressing ΔARF5 increases the level of endogenous indolic compounds.

**Supplementary Figure S7.** Effects of repressing auxin biosynthesis in *proXVE:ΔARF5 proDR5:GFP* during somatic embryogenesis induction.

**Supplementary Table S1.** Expression levels of *TAA1, TAR1, YUC3, YUC5,* and *YUC8*.

**Supplementary Table S2.** The primers used in experiments.

## Dive Curated Terms

The following phenotypic, genotypic, and functional terms are of significance to the work described in this paper:

ARF2 Gramene: AT5G62000
ARF2 Araport: AT5G62000

AGL15 Gramene: AT5G13790
AGL15 Araport: AT5G13790

AGL18 Gramene: AT3G57390
AGL18 Araport: AT3G57390

PIF4 Gramene: AT2G43010
PIF4 Araport: AT2G43010

WOX2 Gramene: AT5G59340
WOX2 Araport: AT5G59340

YUC11 Gramene: AT1G21430
YUC11 Araport: AT1G21430

TAA1 Gramene: AT1G70560
TAA1 Araport: AT1G70560

YUC5 Gramene: AT5G43890
YUC5 Araport: AT5G43890

TAR1 Gramene: AT1G23320
TAR1 Araport: AT1G23320

ARF8 Gramene: AT5G37020
ARF8 Araport: AT5G37020

ARF19 Gramene: AT1G19220
ARF19 Araport: AT1G19220

YUC4 Gramene: AT5G11320
YUC4 Araport: AT5G11320

YUC2 Gramene: AT4G13260
YUC2 Araport: AT4G13260

CYP79B2 Gramene: AT4G39950
CYP79B2 Araport: AT4G39950

CYP79B3 Gramene: AT2G22330
CYP79B3 Araport: AT2G22330

GFP Gramene: green fluorescent protein
GFP Araport: green fluorescent protein

BDL Gramene: AT1G04550
BDL Araport: AT1G04550

TIN Gramene: AT4G27090
TIN Araport: AT4G27090

ARF5/MP/MP11ir Gramene: AT1G19850
ARF5/MP/MP11ir Araport: AT1G19850

TAR2 Gramene: AT4G24670
TAR2 Araport: AT4G24670

YUC1 Gramene: AT4G32540
YUC1 Araport: AT4G32540

YUC3 Gramene: AT1G04610
YUC3 Araport: AT1G04610

YUC6 Gramene: AT5G25620
YUC6 Araport: AT5G25620

YUC7 Gramene: AT2G33230
YUC7 Araport: AT2G33230

YUC8 Gramene: AT4G28270
YUC8 Araport: AT4G28270

YUC9 Gramene: AT1G04180
YUC9 Araport: AT1G04180

YUC10 Gramene: AT1G48910
YUC10 Araport: AT1G48910
