# Supplementary material for: MONOPTEROS isoform MP11ir plays a role during somatic embryogenesis in Arabidopsis thaliana
Source: Plant Physiol. 2025 Nov 20;199(4):kiaf602. doi: 10.1093/plphys/kiaf602 (PMC12671496; doi:10.1093/plphys/kiaf602)
Supplement: kiaf602_Supplementary_Data [file kiaf602_supplementary_data.pdf]

Supplementary material

MONOPTEROS isoform MP11ir plays a role during somatic embryogenesis in *Arabidopsis thaliana*

Barbara Wójcikowska[1,2 # *], Samia Belaidi[1,3#], Victoria Mironova[4], Sylvie Citerne[5], Hélène S. Robert[1*]

[1] Hormonal Crosstalk in Plant Development, Mendel Centre for Plant Genomics and Proteomics, CEITEC MU - Central European Institute of Technology, Masaryk University, Brno, Czech Republic

[2] Institute of Biology, Biotechnology and Environmental Protection, Faculty of Natural Sciences, University of Silesia in Katowice, Katowice, Poland

[3] Laboratory of Functional Genomics and Proteomics, National Centre for Biomolecular Research, Faculty of Science, Masaryk University, Brno, Czechia

[4] Department of Plant and Animal Biology, Radboud Institute for Biological and Environmental Sciences (RIBES), Radboud University, The Netherlands

[5] Université Paris-Saclay, INRAE, AgroParisTech, Institute Jean-Pierre Bourgin for Plant Sciences (IJPB), 78000, Versailles, France.

# These authors contributed equally to this work.

* Authors to whom correspondence should be addressed (barbara.wojcikowska@us.edu.pl and helene.robert.boisivon@ceitec.muni.cz).

Supplementary Table S1. Expression levels of *TAA1, TAR1, YUC3, YUC5,* and *YUC8*. Supporting information for Figure 5. Data indicate the relation expression values ± SD as specified in the legend of Figure 5

Line	Day	*TAA1*	*TAR1*	*YUC3*	*YUC5*	*YUC8*
proXVE:ΔARF5 proDR5:GFP	**1d**	1.74±0.09	27.83±16.03	1.04±0.45	11.69±5.87	16.58±6.22
	**5d**	0.83±0.11	0.92±0.43	2.20±0.12	1.21±0.35	5.75±0.69
	**10d**	1.46±0.25	0.73±0.61	0.46±0.05	2.47±0.54	0.97±0.17
pro35S:bdl-GR	**5d**	0.22±0.05	0.04±0.03	0.03±0.03	1.70±0.26	0.10±0.02
	**10d**	0.23±0.11	0.05±0.04	0.03±0.02	2.43±1.16	0.13±0.08
mpS319	**0d**	0.56±0.34	0.04±0.13	0.17±0.14	0.34±0.19	0.04±0.01
	**10d**	0.20±0.16	0.05±0.17	0.32±0.25	0.09±0.05	0.25±0.15
mpS319	**5d**	0.15±0.07	0.12±0.09	0.02±0.03	0.02±0.02	0.05±0.08
	**10d**	0.20±0.16	0.65±0.17	0.32±0.25	0,09±0,05	0.25±0.15
proMP:MP mpS319	**5d**	1.25±0.30	0.95±0.60	1.63±0.55	0.95±0.57	1.12±0.70
	**10d**	0.83±0.38	0.48±0.15	1.40±0.24	3,55±1,60	0.88±0.32
proMP:MP11ir mpS319	**5d**	1.62±0.07	0.86±0.50	0.53±0.07	0.68±0.69	0.61±0.26
	**10d**	0.64±0.06	0.11±0.10	0.67±0.16	2,14±0,81	0.35±0.07
proMP:MP11ir proMP:MP mpS319	**5d**	1.03±0.38	0.30±0.19	1.40±0.48	1.28±0.83	0.98±0.56
	**10d**	1.30±0.32	0.26±0.23	2.35±0.12	1,79±1,33	1.46±0.29

Supplementary Table S2. The primers used in experiments.

**Primers used in RT-qPCR/PCR analysis of *TF* and *YUC* genes**				
**Gene**	**ID**	**pF 5'-3'**	**pR 5'-3'**	**Publication**
MP11ir	*AT1G19850*	TGGGTAATGTTTTGACTTGG	CCACAAACTCTTCCCATGGAT	Cucinotta et al., 2021
MP	*AT1G19850*	CACTAAGGTTCAAAAAACCG	TTACGCATCCCACAAACTCTTCC	Wójcikowska and Gaj, 2017
TIN	*AT4G27090*	GTCGTTATCGTCGACGTTGTT	CCTCGATCAAAGCCTTCTTCT	Wójcikowska et al., 2013
TAA1	*AT1G70560*	TTCGTGGTCAATCTGGATCATGG	ACCACGTATCGTCACCGTACAC	
TAR1	*AT1G23320*	CGCAGCGGTTCCTTATTACTCCAC	TTGTCGAACGTCCTTGCGTCTC	Present work
TAR2	*AT4G24670*	GCTCTTCACTGCTTCAAAGAGCAC	TCTGTCTTTCACCAAAGCCCATCC	
YUC1	*AT4G32540*	CGGAACACCGTTCATGTGT	CCGGTGACATTTTTCAGCTC	Wójcikowska et al., 2013
YUC2	*AT4G13260*	TTGTGGTTCGTGACTCGGTA	TTCAAGAGGGCCAAGTTTTG	
YUC3	*AT1G04610*	GATGGCCGTGTTCTTGAGAT	TCATGAGCCACACTCATAGC	
YUC4	*AT5G11320*	AACTCCCGTTCTTGATGTCG	AAAAACTATTCTCCTTAAGCCAATC	
YUC5	*AT5G43890*	TGTCCAGTCTGCTCGATACG	TTCTCGCCGGATTTGTACTC	
YUC6	*AT5G25620*	GGTAAAACTCCGGTTCTCGAC	TTGGAAATCCATCTTTCTTACTAAAC	
YUC7	*AT2G33230*	TGAAGAACACCGCAGGTAAA	CCAAGTCGTTTTCCTTAAGCC	
YUC8	*AT4G28720*	CGTCTCAAGCTTCACCTTCC	AGCCACTGGTCTCATCGAAC	
YUC9	*AT1G04180*	TGGTCGTTAGAAGCTCGGTT	CGGCGTCTTTCCTGTCAT	
YUC10	*AT1G48910*	TTACCGGAAAAGCTCCTGTC	TCACGTATTCATAGTCCTCTAACCA	
YUC11	*AT1G21430*	GAGAATGGCGAAGGTGTGAT	TAACACGTGCACCTGGCTAC	

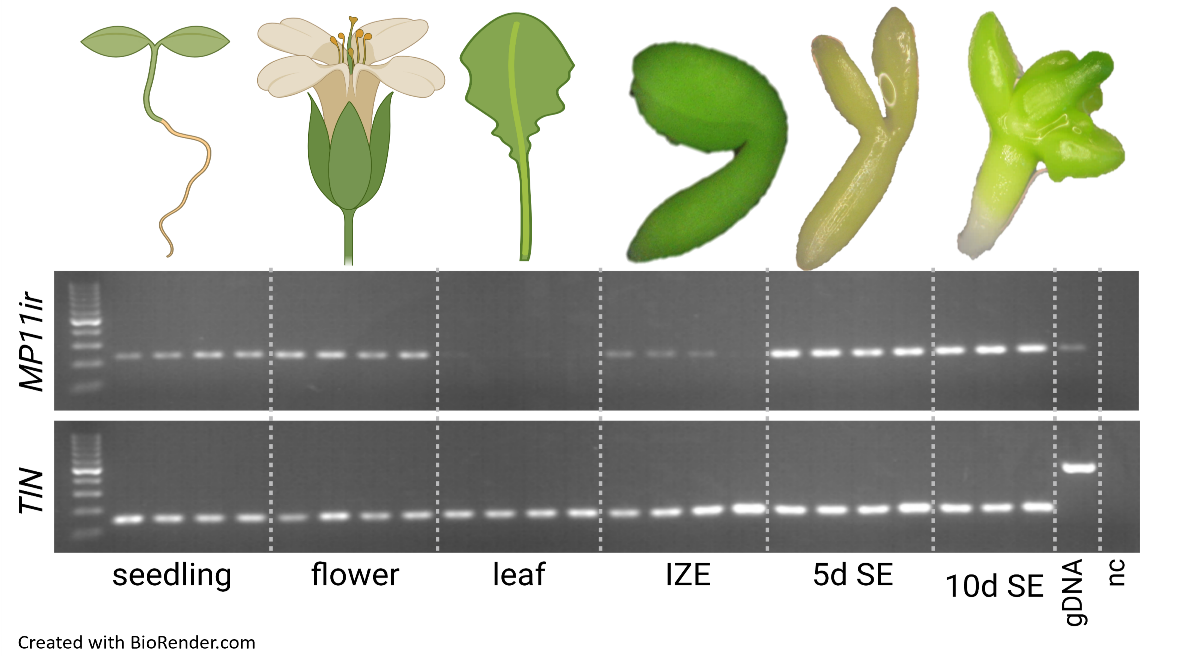

Supplementary Figure S1. ***MP11ir*** **is expressed during somatic embryogenesis.** Presence of *MP11ir* transcript during plant development *in vivo* and auxin-dependent SE induction *in vitro* (5.0 µM 2,4-D). IZE – immature zygotic embryo; SE – somatic embryogenesis; 5d – 5th day of SE induction; 10d – 10th day of SE induction; gDNA – genomic DNA; nc – negative control; *TIN* – reference gene. Created with BioRender.com.

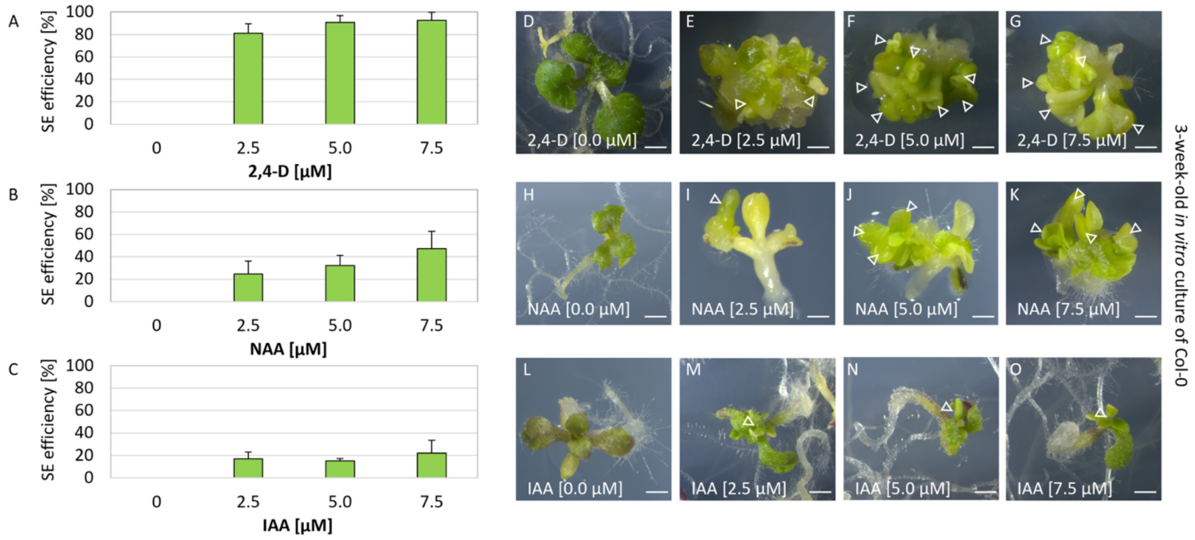

Supplementary Figure S2. The induction of somatic embryogenesis depends on 2,4-D and NAA, but not IAA.

The capacity of IZE explants cultured for three weeks on media with different 2,4-D (**A, D-G**), NAA (**B, H-K**), and IAA (**C, L-O**) concentrations: 0.0 (**D, H, L**), 2.5 (**E, I, M**), 5.0 (**F, J, N**), and 7.5 µM (**G, K, O**); (n = 3; means ± SD are presented). IZE explants cultured on auxin-free medium developed seedlings after three weeks of culture (**D, H, L**). The increasing embryogenic capacity of the Col-0 IZE explants cultured for three weeks on media with different 2,4-D or NAA concentrations (2.5 (**E, I**), 5.0 (**F, J**), and 7.5 µM (**G, K**)). The weak embryogenic capacity of the Col-0 IZE explants cultured for three weeks on media with different IAA concentrations (2.5 (**M**), 5.0 (**N**), and 7.5 µM (**O**)). The white arrowhead indicates the somatic embryo. Size bars indicate 1 mm. 2,4-D – 2,4-dichlorophenoxyacetic acid; IAA – indole-3-acetic acid; IZE – immature zygotic embryo; NAA – 1-naphthaleneacetic acid.

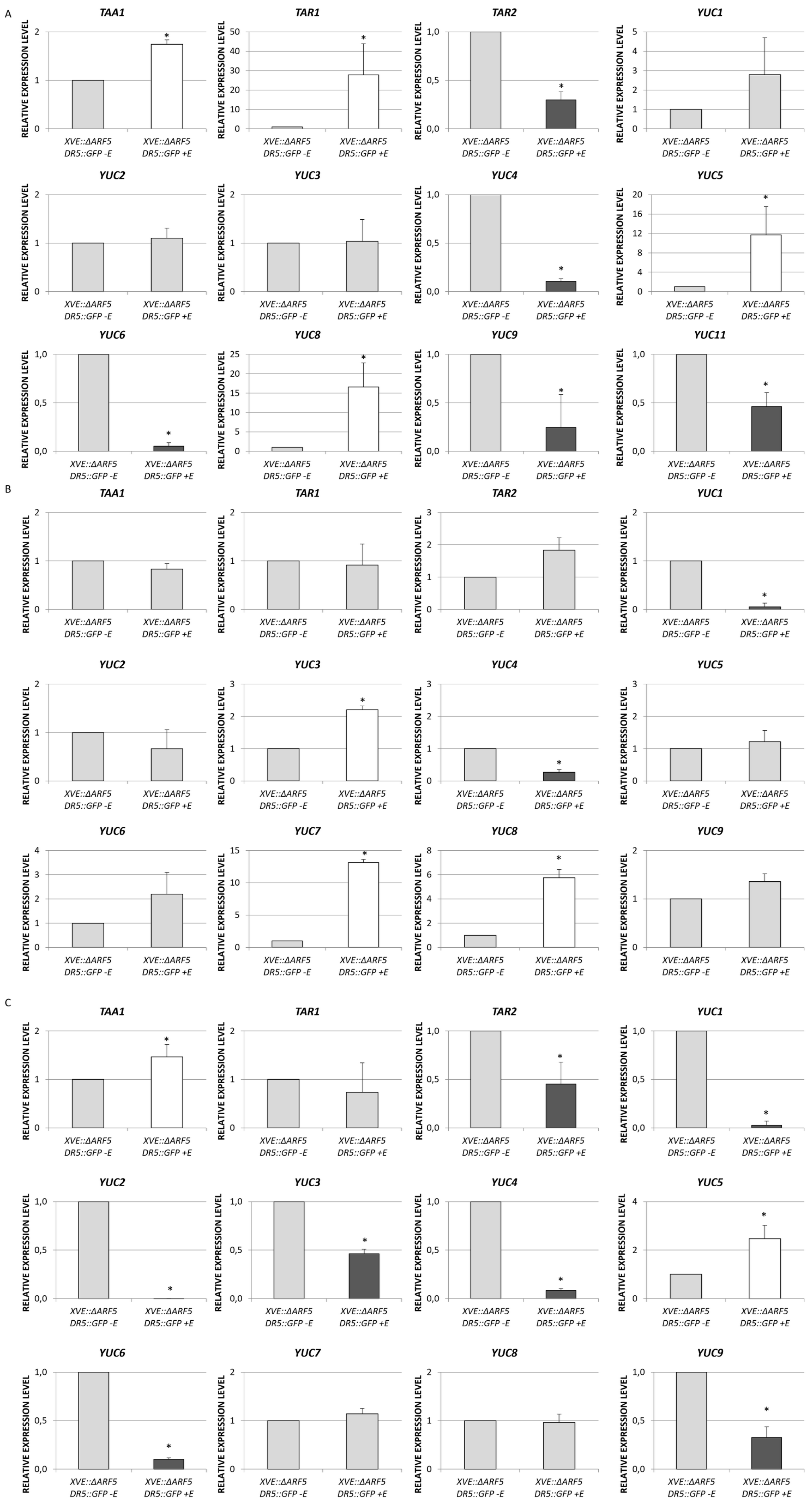
A
TAA1
TAR1
TAR2
YUC1
YUC2
YUC3
YUC4
YUC5
YUC6
YUC8
YUC9
YUC11
B
TAA1
TAR1
TAR2
YUC1
YUC2
YUC3
YUC4
YUC5
YUC6
YUC7
YUC8
YUC9
C
TAA1
TAR1
TAR2
YUC1
YUC2
YUC3
YUC4
YUC5
YUC6
YUC7
YUC8
YUC9
RELATIVE EXPRESSION LEVEL
XVE::ΔARF5 DR5::GFP -E
XVE::ΔARF5 DR5::GFP +E
*

Supplementary Figure S3. Expression level of auxin biosynthetic genes in *proXVE:ΔARF5 proDR5:GFP* somatic embryos.

(**A-C**) Relative expression level of auxin biosynthesis genes in the 1- (**A**), 5- (**B**), and 10-day-old (**C**) embryogenic cultures of the *proXVE:ΔARF5 proDR5:GFP* transgenic line treated with 1 µM β-estradiol (+E). The relative expression level was normalized to internal control (*AT4G27090*) and calibrated to the *proXVE:ΔARF5 proDR5:GFP* culture of the same age and untreated with 1 µM β-estradiol (-E). Value significantly different from the *proXVE:ΔARF5 proDR5:GFP* (-E) culture of the same age is marked with asterisks (Student's t-test, *$p < 0.05$; n = 3; means ± SD are presented). Gray bars indicate no statistical differences, white bars indicate that the gene is upregulated when *ΔARF5* is induced, and black bars when the gene expression is reduced.

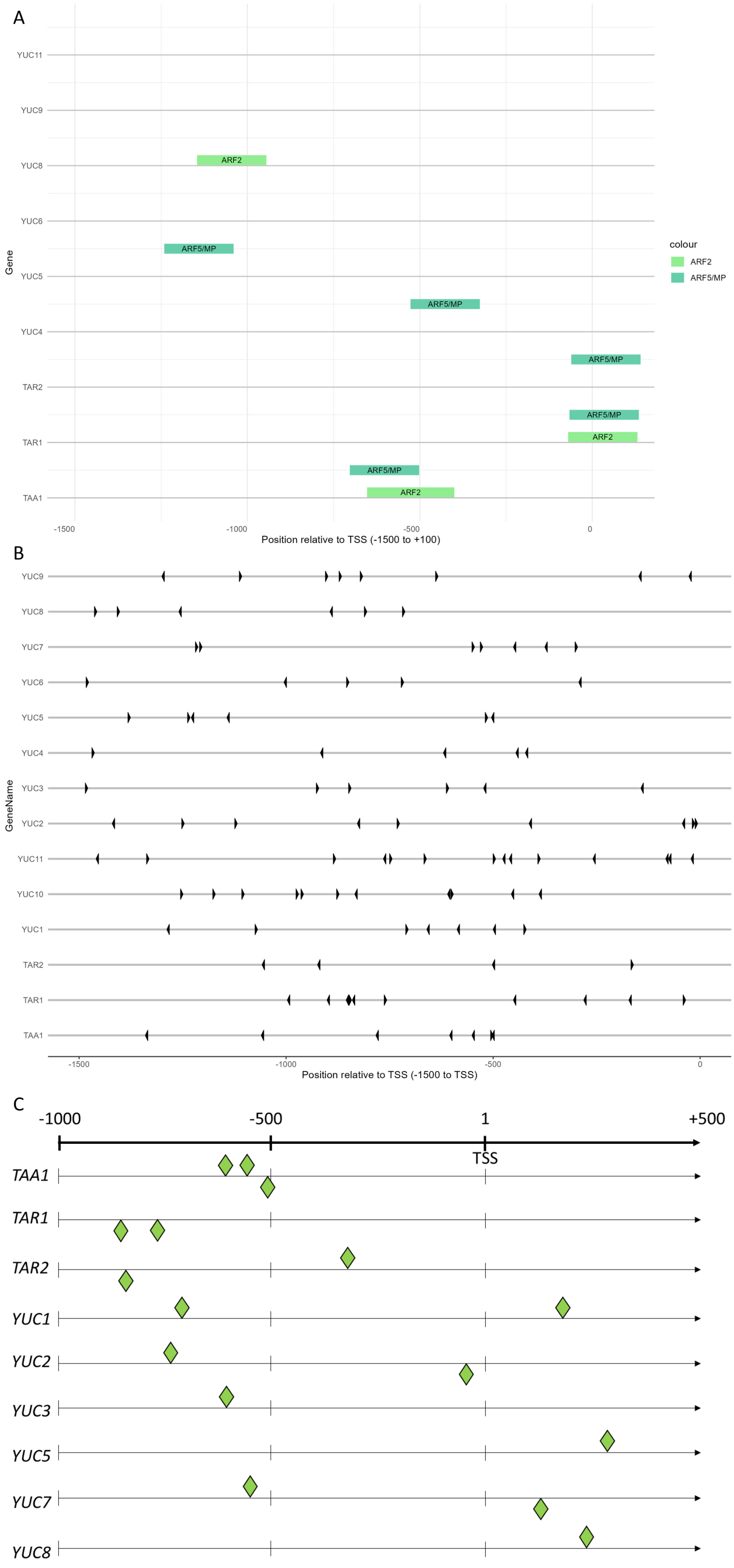
A
YUC11
YUC9
YUC8
YUC6
YUC5
YUC4
TAR2
TAR1
TAA1
Gene
ARF2
ARF5/MP
colour
-1500
-1000
-500
0
Position relative to TSS (-1500 to +100)
B
YUC9
YUC8
YUC7
YUC6
YUC5
YUC4
YUC3
YUC2
YUC11
YUC10
YUC1
TAR2
TAR1
TAA1
GeneName
-1500
-1000
-500
0
Position relative to TSS (-1500 to TSS)
C
-1000
-500
1
+500
TSS
TAA1
TAR1
TAR2
YUC1
YUC2
YUC3
YUC5
YUC7
YUC8

Supplementary Figure S4. Analysis of the *TAA1*, *TAR1*, and *YUC* promoters for MP binding sites.

(**A**) The *TAA1*, *TAR*, and *YUC* gene promoters were analyzed for MP and ARF2 binding sites. (**B**) Binding regions detected by DAP-seq in (O'Malley et al., 2016) TGTCnn sequences. (**C**) The presence of AuxRe element based on the PlantPan 4.0 database.

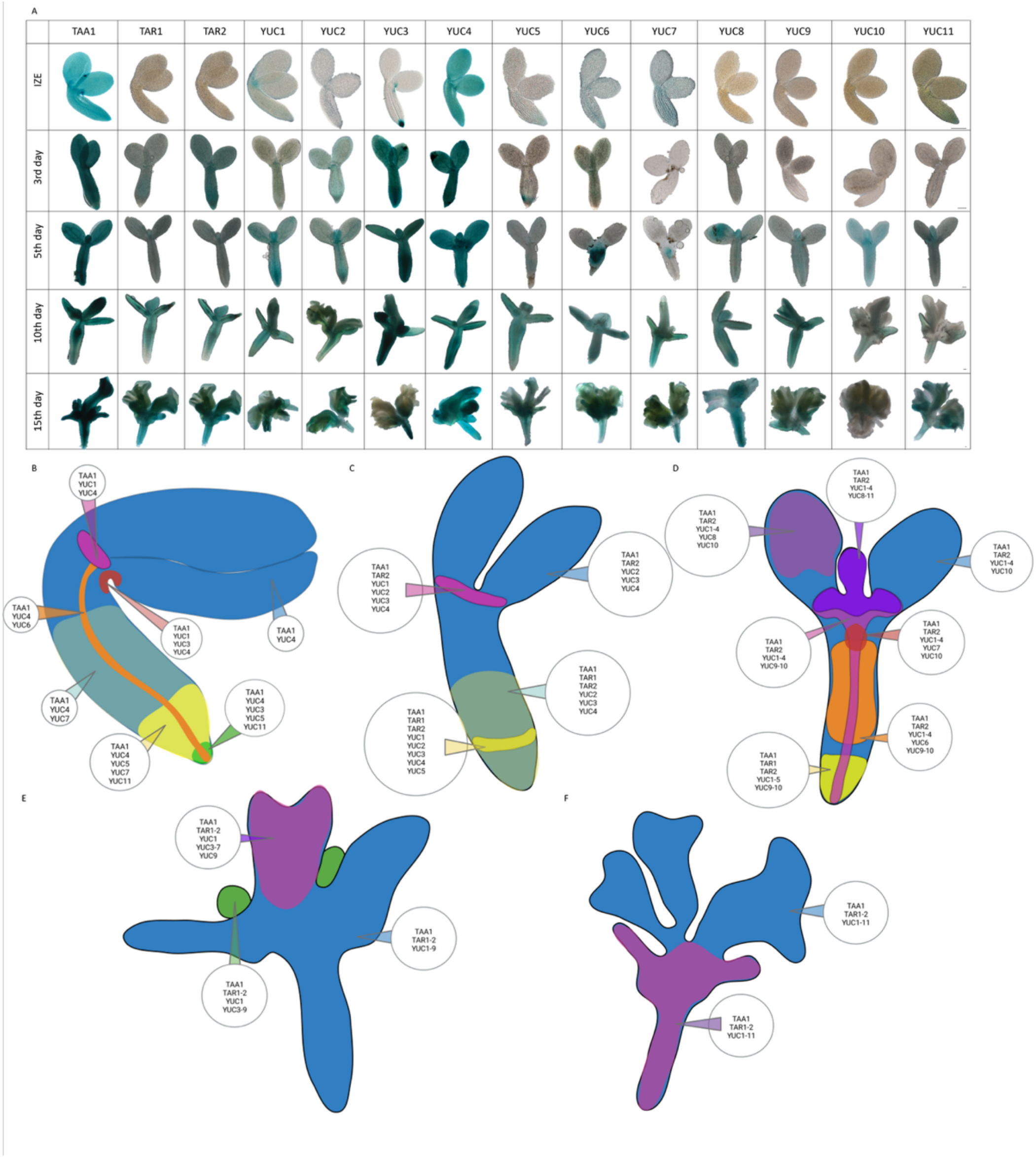

Supplementary Figure S5. Expression pattern of TAA1, TAR1, TAR2, and YUC1-11 during somatic embryogenesis induction.

(**A**) Spatio-temporal localization of TAA1, TAR1, TAR2, and YUC1-11 enzymes during SE process under *in vitro* culture. (**B-F**) Map of TAA1, TAR1, TAR2, YUC1-11 enzymes involved in auxin biosynthesis in IZE explants of reporter lines (**B**), and explants cultured on E5 medium and on 3- (**C**), 5- (**D**), 10- (**E**), 15-day-old (**F**) *in vitro* culture. Images were digitally extracted for comparison. Size bars indicate 100 µm (**A**). Summary drawings were prepared using BioRender. IZE – immature zygotic embryo; SE – somatic embryogenesis.

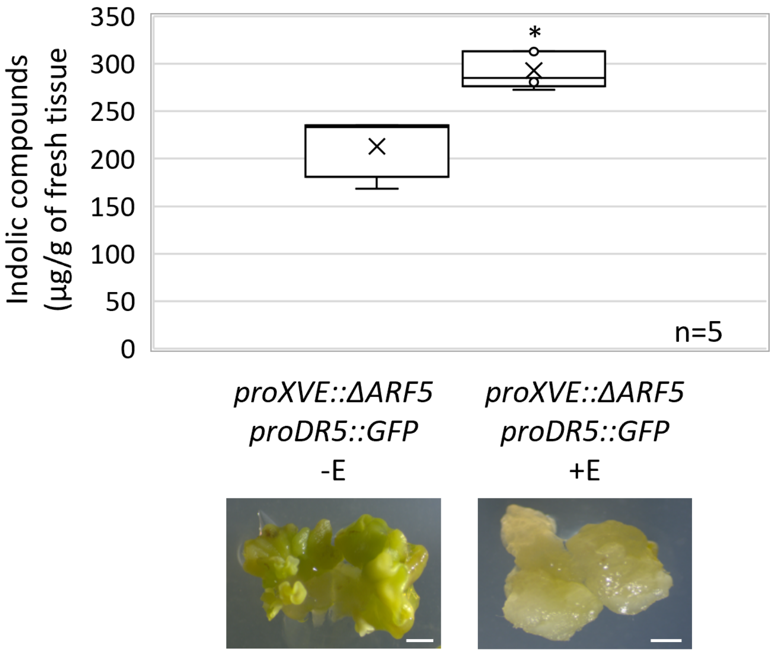

Figure S6. Overexpressing *ΔARF5* increases the level of endogenous indolic compounds. Level of indolic compounds (µg/g of fresh tissue) in 3-week-old IZE-culture of *proXVE:ΔARF5 proDR5:GFP* transgenic line with 1 µM β-estradiol-induced *ΔARF5* overexpression. Size bars indicate 1 mm. The data are presented as a boxplot. The cross represents the mean value; the center line represents the median value; the box limits are upper and lower quartiles; the whiskers show the 1.5x interquartile range; the open circles are outliers. The value in *proXVE:ΔARF5 proDR5:GFP* (+E) is significantly different from the *proXVE:ΔARF5 proDR5:GFP* (-E) culture of the same age is marked with asterisks (*$p < 0.05$; n = 5; Student's t-test). The images illustrate the type of samples and are identical to those used in Figure 4C, bottom row. IZE – immature zygotic embryo.

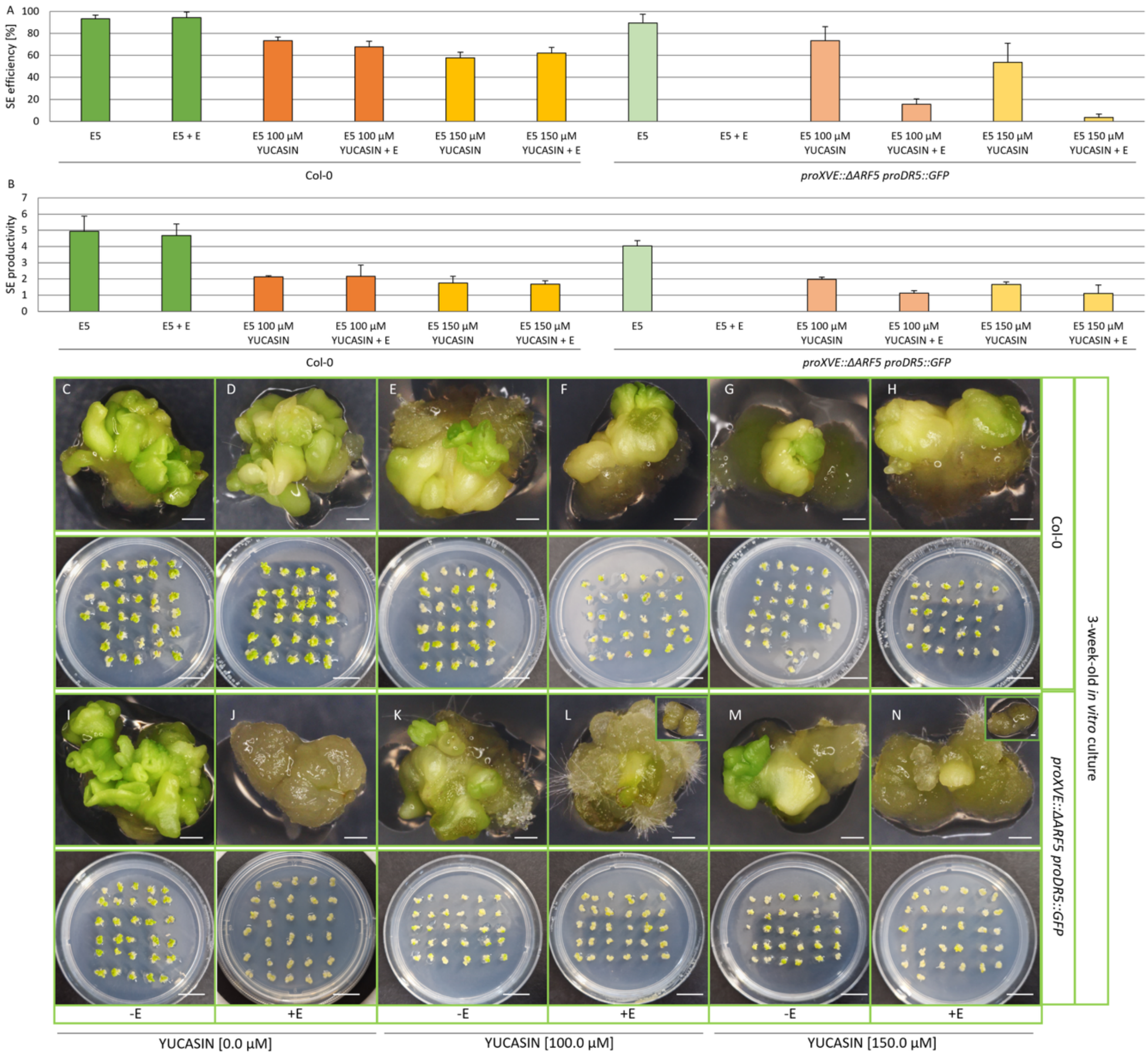

Supplementary Figure S7. Effects of repressing auxin biosynthesis in *proXVE:ΔARF5 proDR5:GFP* during somatic embryogenesis induction.

The effect of auxin biosynthesis inhibitor yucasin on the SE efficiency (**A**) and productivity (**B**) of the Col-0 (**C-H**) and *proXVE:ΔARF5 proDR5:GFP* (**I-N**) IZE explants. The capacity of Col-0 and *proXVE:ΔARF5 proDR5:GFP* IZE explants cultured for three weeks on E5 media with different yucasin concentrations: 0 (**C, D, I, J**), 100 (**E, F, K, L**), and 150 µM (**G, H, M, N**), supplemented (**D, F, H, J, L, N**) or not (**C, I, E, K, G, M**) with 1 µM estradiol (+E) for induction of *ΔARF5* overexpression; (n = 3; means ± SD are presented). A representative image of the plates is shown in the bottom row. A representative image of a somatic embryo is shown in the top row. In insets (**L, N**), alternative phenotypes are shown. Col-0 IZE explants cultured on control media (E5 and E5 + E) developed numerous somatic embryos after three weeks of culture (**C, D**). Different yucasin concentrations resulted in the decreasing embryogenic capacity of the Col-0 IZE explants cultured for three weeks on E5 and E5 + E media (**A, B, E-H**). The embryogenic capacity of the *proXVE:ΔARF5 proDR5:GFP* IZE explants cultured for three weeks on E5 media (**A, B, I**) is comparable to Col-0 IZE explants (**C**). Explants with *ΔARF5*-overexpression (E5 + E) lack a SE response (**J**). Different yucasin concentrations resulted in a decrease in the embryogenic capacity of the *proXVE:ΔARF5 proDR5:GFP* IZE explants cultured for three weeks on E5 media with (**A, B, K, M**). Explants overexpressing

ΔARF5 (+E) cultured on E5 media supplemented with yucasin at different concentrations were capable, but with very low efficiency and productivity, to regenerate somatic embryos. They mostly produced callus (**A, B, L, N**). Size bars indicate 1 mm for explant images and 1 cm for plate images. E – estradiol; IZE – immature zygotic embryo; SE – somatic embryogenesis.
